# Free-water: A promising structural biomarker for cognitive decline in aging and mild cognitive impairment

**DOI:** 10.1162/imag_a_00293

**Published:** 2024-09-18

**Authors:** Aditi Sathe, Yisu Yang, Kurt G. Schilling, Niranjana Shashikumar, Elizabeth Moore, Logan Dumitrescu, Kimberly R. Pechman, Bennett A. Landman, Katherine A. Gifford, Timothy J. Hohman, Angela L. Jefferson, Derek B. Archer

**Affiliations:** Vanderbilt Memory and Alzheimer’s Center, Vanderbilt University School of Medicine, Nashville, TN, United States; Vanderbilt University Institute of Imaging Science, Vanderbilt University Medical Center, Nashville, TN, United States; Department of Medicine, Vanderbilt University Medical Center, Nashville, TN, United States; Vanderbilt Genetics Institute, Vanderbilt University Medical Center, Nashville, TN, United States; Department of Biomedical Engineering, Vanderbilt University, Nashville, TN, United States; Department of Electrical and Computer Engineering, Vanderbilt University, Nashville, TN, United States; Department of Radiology & Radiological Sciences, Vanderbilt University Medical Center, Nashville, TN, United States; Department of Neurology, Vanderbilt University Medical Center, Nashville, TN, United States

**Keywords:** Alzheimer’s disease (AD), diffusion MRI, free-water, fractional anisotropy (FA), cognition, aging

## Abstract

Diffusion MRI derived free-water (FW) metrics show promise in predicting cognitive impairment and decline in aging and Alzheimer’s disease (AD). FW is sensitive to subtle changes in brain microstructure, so it is possible these measures may be more sensitive than traditional structural neuroimaging biomarkers. In this study, we examined the associations among FW metrics (measured in the hippocampus and two AD signature meta-ROIs) with cognitive performance, and compared FW findings to those from more traditional neuroimaging biomarkers of AD. We leveraged data from a longitudinal cohort (n_participants_ = 296, n_observations_ = 870, age at baseline: 73 ± 7 years, 40% mild cognitive impairment [MCI]) of older adults who underwent serial neuropsychological assessment (episodic memory, information processing speed, executive function, language, and visuospatial skills) and brain MRI over a maximum of four time points, including baseline (n = 284), 18-month (n = 246), 3-year (n = 215), and 5-year (n = 125) visits. The mean follow-up period was 2.8 ± 1.3 years. Structural MRI was used to quantify hippocampal volume, in addition to Schwarz and McEvoy AD Signatures. FW and FW-corrected fractional anisotropy (FAFWcorr) were quantified in the hippocampus (hippocampal FW) and the AD signature areas (Schwarz_FW_, McEvoy_FW_) from diffusion-weighted (dMRI) images using bi-tensor modeling (FW elimination and mapping method). Linear regression assessed the association of each biomarker with baseline cognitive performance. Additionally, linear mixed-effects regression assessed the association between baseline biomarker values and longitudinal cognitive performance. A subsequent competitive model analysis was conducted on both baseline and longitudinal data to determine how much additional variance in cognitive performance was explained by each biomarker compared to the covariate only model, which included age, sex, race/ethnicity, apolipoprotein-ε4 status, cognitive status, and modified Framingham Stroke Risk Profile scores. All analyses were corrected for multiple comparisons using an FDR procedure. Cross-sectional results indicate that hippocampal volume, hippocampal FW, Schwarz and McEvoy AD Signatures, and the Schwarz_FW_ and McEvoy_FW_ metrics are all significantly associated with memory performance. Baseline competitive model analyses showed that the McEvoy AD Signature and Schwarz_FW_ explain the most unique variance beyond covariates for memory (ΔR_adj_^2^ = 3.47 ± 1.65%) and executive function (ΔR_adj_^2^ = 2.43 ± 1.63%), respectively. Longitudinal models revealed that hippocampal FW explained substantial unique variance for memory performance (ΔR_adj_^2^ = 8.13 ± 1.25%), and outperformed all other biomarkers examined in predicting memory decline (p_FDR_ = 1.95 x 10^-11^). This study shows that hippocampal FW is a sensitive biomarker for cognitive impairment and decline, and provides strong evidence for further exploration of this measure in aging and AD.

## 1 Introduction

Alzheimer’s disease (AD) is the most common form of dementia, currently affecting over 6.7 million people in the US alone and expected to climb rapidly to 13.8 million by 2060 (2023 Alzheimer’s Disease Facts and Figures, 2023). At particularly high risk of developing AD are individuals with mild cognitive impairment (MCI)—a transitional stage between normal aging and dementia (Morris et al., 2001). It is crucial to identify predictive biomarkers of disease progression to facilitate early diagnosis and treatment of AD.

AD is characterized pathologically by the extracellular accumulation of beta-amyloid neurite plaques and intracellular formation of phosphorylated tau neurofibrillary tangles (Crews & Masliah, 2010; Perl, 2010). The neuropathology-driven gray matter atrophy typically starts in the medial temporal lobe structures (e.g., entorhinal cortex, hippocampus) and then spreads to other regions of the brain (Braak et al., 2008; Coupé et al., 2019). This pattern of deterioration is well supported by studies investigating the relationship of medial temporal lobe atrophy with cognitive decline and AD diagnostic status (Aisen et al., 2017; Braskie & Thompson, 2013; Chino et al., 2022). For instance, hippocampal volume has been found to distinguish cognitively unimpaired (CU) controls from participants with MCI (Apostolova et al., 2012) or AD^10^, (Pennanen et al., 2004) and MCI from AD^11^ participants. Moreover, hippocampal atrophy observed in MCI participants at baseline has been associated with later conversion to AD dementia (Jack et al., 1999). For these reasons, hippocampal volume has been appropriately identified as the primary structural neuroimaging biomarker for diagnosing AD and monitoring disease progression (Jack et al., 2018; Sperling et al., 2011).

More recent studies suggest that regional cortical thickness measures are also sensitive predictors of AD diagnostic status and cognitive decline (Dickerson et al., 2009). Specifically, increased cortical thinning in AD-vulnerable regions of interest (ROIs) (e.g., entorhinal cortex, lateral temporal cortex, inferior parietal cortex) is associated with more advanced AD clinical stages (Bakkour et al., 2009) and a higher risk of progressing to more severe disease stages (e.g., CU to MCI, MCI to AD) (Pettigrew et al., 2023). AD-specific cortical thinning is also shown to predict cognitive decline (Dickerson & Wolk, 2012; Sabuncu et al., 2011) and AD-related neuropathology (i.e., amyloid or tau positivity) (Pettigrew et al., 2023). Therefore, the use of cortical thickness measurements combined from multiple regions into an “AD Signature” meta-ROI has been recommended (Dickerson et al., 2009; Dickerson & Wolk, 2012; Ortiz et al., 2014).

McEvoy et al. (2009) conducted a systematic evaluation of regional volume reductions as a potential biomarker for AD and established a novel AD signature based on both cortical thickness and hippocampal volume measurements. This signature used ROI-specific weights to appropriately reflect the differential rates of atrophy across regions and discriminated between CU controls and AD patients with high sensitivity (83%) and specificity (93%). A large-scale comparison of cortical thickness and volume methods was also performed by Schwarz et al. (2016) where they proposed a novel thickness-based AD signature that exhibited a strong correlation with AD pathology, predicted AD diagnostic status, and did not require total intracranial volume (TIV) correction.

Diffusion magnetic resonance imaging (dMRI) enables the *in vivo* quantification of microstructural changes in the brain and may provide more sensitivity than the macrostructural volume measurements of traditional MRI to predict clinical decline (Kantarci et al., 2005; Müller et al., 2005; Ringman et al., 2007). Traditionally, diffusion tensor imaging (DTI) has been used to assess white matter microstructure (Bozzali, Falini, et al., 2002), and its utility as a biomarker for distinguishing between MCI and AD has been evaluated (Stebbins & Murphy, 2009). For example, decreased fractional anisotropy (FA) (Takahashi et al., 2002; Y. Zhang et al., 2007) and elevated diffusivity (Wang et al., 2012; Weston et al., 2015) have been consistently found in prodromal AD and cognitively normal individuals who develop MCI. Although extensive research has used dMRI to evaluate the role of white matter in AD (Caballero et al., 2017; Stone et al., 2021), less emphasis has been placed on using dMRI to assess gray matter microstructure (Bozzali, Cercignani, et al., 2002; Weston et al., 2015).

Despite its vital contributions, a major drawback of conventional DTI metrics is their well-established susceptibility to partial volume effects (Alexander et al., 2001; Vos et al., 2011), whereby the conventional FA (FA_CONV_) map fails to distinguish between tissue and fluid compartments within each voxel (Metzler-Baddeley et al., 2012) and becomes confounded in its discriminatory power (Berlot et al., 2014). This issue can be addressed with advanced post-processing techniques like free-water [FW] correction (Pasternak et al., 2009). FW correction separates the FA_CONV_ metric into the intracellular FW-corrected FA (FA_FWcorr_) and extracellular FW measures, largely enhancing biological specificity (Bergamino et al., 2021). The FW index has been proposed as a standalone biomarker of AD-related pathology (Maillard et al., 2022) and may provide more enhanced associations with endophenotypes of AD (Ji et al., 2017). Notably, elevated FW levels at baseline have been associated with AD-related diagnoses (MCI, AD) (Dumont et al., 2019), lower episodic memory and executive function performance at baseline (Maillard et al., 2019), as well as accelerated rates of cognitive decline (Berger et al., 2022). Furthermore, recent work from our group has demonstrated that FW and FA_FWcorr_ are more sensitive to progression in abnormal aging and distinguishes diagnoses along the AD clinical continuum (Archer et al., 2020, 2023; Yang et al., 2023); Ofori et al*.* (2019) also found that hippocampal FW provides unique information about neurodegenerative changes in AD. Together, these findings advance the potential benefit of including FW-corrected DTI metrics as additional biomarkers for AD disease monitoring.

The present study leveraged neuroimaging data from a longitudinal cohort (n_participants_ = 296; n_observations_ = 870) of older adults to investigate the relationships between various AD-related neuroimaging biomarkers and cognition. We hypothesized that FW-corrected measures within these AD-related regions would provide more sensitive associations with cognitive impairment and decline as compared to traditional structural and thickness measures.

## 2 Methods

### 2.1 Study cohort

The Vanderbilt Memory & Aging Project (VMAP) (Jefferson et al., 2016) is a longitudinal observational study investigating vascular health and brain aging, including participants aged 60+ years who are considered CU or have MCI. MCI determinations were based upon National Institute on Aging/Alzheimer’s Association Workgroup core clinical criteria (Albert et al., 2011). VMAP participants (n_participants_ = 296, n_observations_ = 870, age at baseline: 73 ± 7 years, 40% MCI) underwent longitudinal neuropsychological assessment and brain MRI over a maximum of four time points, including baseline (n = 284), 18-month (n = 246), 3-year (n = 215), and 5-year (n = 125) visits. The mean follow-up period was 2.8 ± 1.3 years. At study entry, participants completed a comprehensive evaluation, including fasting blood draw, physical examination, clinical interview with medication review, echocardiogram, and brain MRI (Moore et al., 2021). As part of the extensive screening, participants were excluded for a cognitive diagnosis of dementia, magnetic resonance imaging (MRI) contraindication, history of neurological disease (e.g., multiple sclerosis, stroke), heart failure, major psychiatric illness, head injury with loss of consciousness >5 minutes, or a systemic or terminal illness affecting follow-up participation. Informed consent was provided by all participants, and the Vanderbilt Institutional Review Board approved the protocol. Several demographic and clinical covariates were required for inclusion in the present study, including age, sex, educational attainment, race/ethnicity, apolipoprotein E (*APOE*) carrier status (ε2, ε3, ε4), Framingham Stroke Risk Profile (FSRP; excluding points for age), and cognitive status at baseline (CU, MCI).

### 2.2 Neuropsychological assessment

Participants completed a common, comprehensive neuropsychological protocol assessing language, information processing speed, executive function, visuospatial skills, and episodic learning and memory. As previously described (Kresge et al., 2018), we created psychometrically sound composite measures for memory and executive function. The memory composite compiled scores from the California Verbal Learning Test-Second Edition (CVLT-II) Total Learning, Interference Condition, Long Delay Free Recall, and Recognition components, together with identical components of the Biber Figure Learning Test (Gifford et al., 2020; Glosser et al., 2002). For the executive functioning composite, the Delis-Kaplan Executive Function System (DKEFS) Tower Test, DKEFS Letter-Number Switching, DKEFS Color-Word Inhibition, and Letter Fluency (FAS) test scores were evaluated. The Animal Naming Test and Boston Naming Test scores were used to quantify language performance, and WAIS-IV Coding, and DKEFS Number Sequencing scores assessed information processing speed. The Hooper Visual Organization Test was used to evaluate visuospatial skills.

### 2.3 T1 and diffusion imaging acquisition

Participants were scanned at study entry at the Vanderbilt University Institute of Imaging Science on a 3 T Philips Achieva system (Best, The Netherlands). Imaging parameters have been previously outlined (Jefferson et al., 2016; Moore et al., 2018). Briefly, T1-weighted (fast field echo, repetition time = 8.9 ms, echo time = 4.6 ms, spatial resolution = 1mm isotropic) and dMRI (spin-echo echo-planar imaging, repetition time = 10 s, echo time = 60 ms, spatial resolution = 2 mm isotropic, b-values: 0, 1,000 s/mm^2^) images were acquired. dMRI images were collected along 32 diffusion gradient vectors and five non-diffusion (B0) weighted images.

### 2.4 Imaging processing

#### 2.4.1 Volumetric MRI analysis

T1 images were post-processed using Multi-Atlas (Landman et al., 2020) (for volumes) and FreeSurfer (for cortical thickness) segmentation methods. For FreeSurfer subcortical segmentation and cortical parcellation, surfaces were manually inspected and corrected for registration, topological, and segmentation defects. After manual correction, images were reprocessed to update the transformation template and segmentation information. Total intracranial volume (TIV) (cm^3^) was calculated together with volumes for specific ROIs, including the middle temporal gyrus, entorhinal cortex, and the fusiform gyrus. These measures were used to calculate the Schwarz (Schwarz et al., 2016) and McEvoy (McEvoy et al., 2009) AD-signatures. Briefly, both Schwarz and McEvoy AD signatures are composites formed from cortical thickness (and hippocampal volume for the latter) measurements of ROIs that best discriminate clinically normal individuals from those with AD.

#### 2.4.2 Hippocampal segmentation

Hippocampal segmentation was carried out using *hippodeep* (Thyreau et al., 2018), a deep-learning appearance model that uses a convolutional neural network to rapidly segment the bilateral hippocampus from a T1 image. *hippodeep* is pre-trained on thousands of images from multiple large cohorts and is therefore quite robust to subject- and MR-contrast variation (Schell et al., 2023; Zavaliangos‐Petropulu et al., 2020). Hippocampal volume was quantified by summing the left and right hippocampal volumes. The hippocampal volume measure was adjusted by TIV based on an established approach (Mormino et al., 2014), which allowed us to eliminate the need for a TIV covariate in our statistical analyses.

#### 2.4.3 Diffusion MRI analysis

The *PreQual* (Cai et al., 2021) pipeline was utilized to preprocess all dMRI data, ensuring robust processing for all dMRI data (e.g., denoising, eddy current correction, motion correction). The quality control PDFs generated by the *PreQual* pipeline underwent manual inspection, and participants with poor data quality were excluded from the analysis. Generally, imaging sessions were removed due to inaccurate synthetic b0 creation, inaccurate brain masking, and excessive motion. The preprocessed data were then used in custom MATLAB code to calculate metrics corrected for FW, including extracellular FW (FW) and FW-corrected fractional anisotropy (FA_FWcorr_) using the bi-tensor modeling framework established (Pasternak et al., 2009). The T1 image from each participant was subsequently down-sampled to 2 mm resolution and co-registered to the corresponding b0 image using FLIRT with nearest-neighbor interpolation (Jenkinson et al., 2002).

#### 2.4.4 Quantifying diffusivity within structural regions

**Fig. 1.**
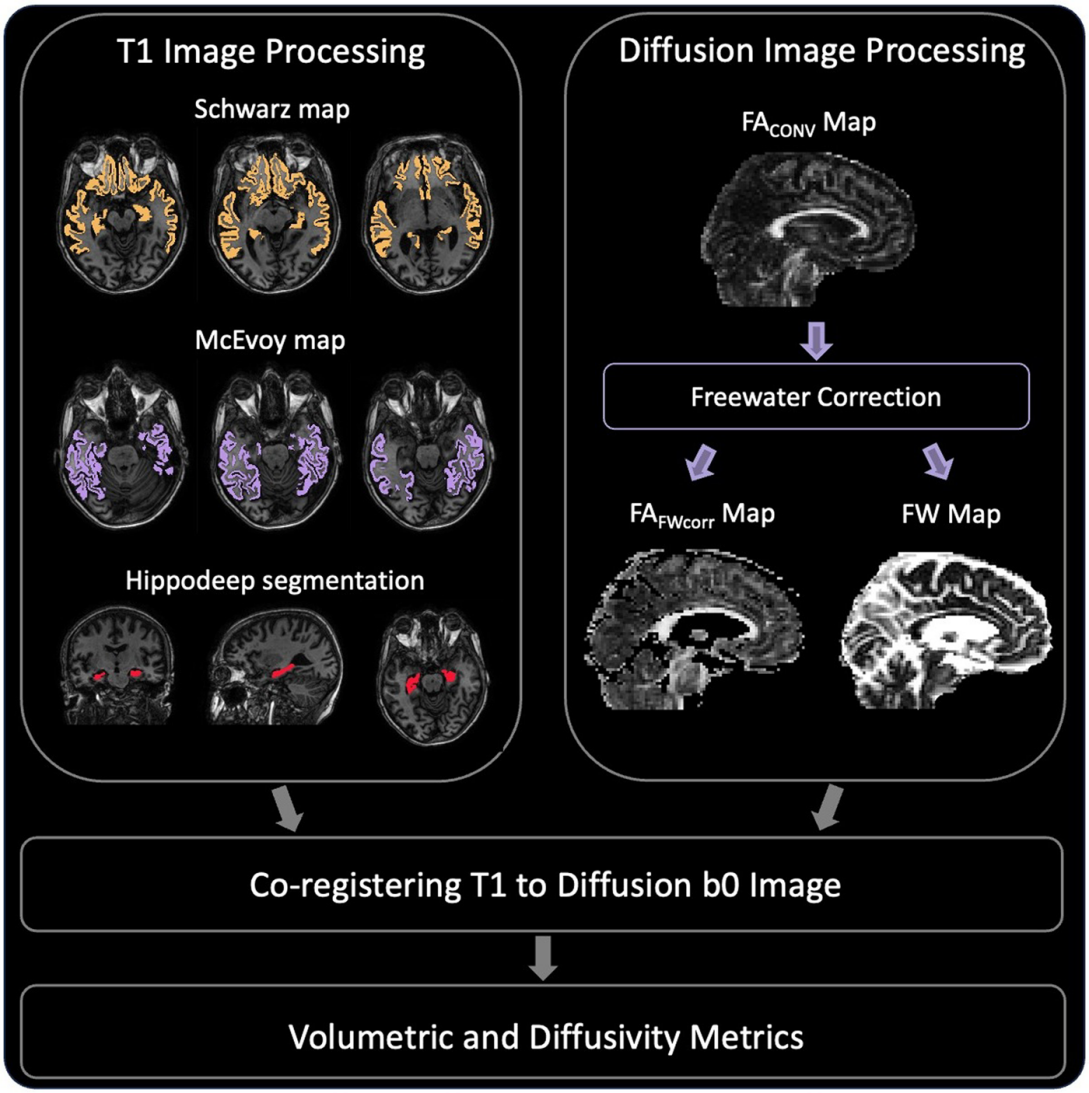
Neuroimaging Workflow. The hippocampus and Schwarz/McEvoy AD Signature area segmentations were obtained from the T1 image; the diffusion image was used to calculate conventional and FW-corrected DTI metrics. Next, the structural images were co-registered to the diffusion b0 image. Biomarkers were then estimated. These steps were performed for the baseline imaging visit for each participant.

An illustration of our neuroimaging workflow is detailed in Figure 1. In addition to using the FreeSurfer and Multiatlas segmentations to calculate the Schwarz/McEvoy AD signature scores, we also overlaid the two meta-ROI segmentations on top of the FW and FA_FWcorr_ maps to quantify diffusivity within these regions. The final AD signature-diffusivity metrics were created by averaging diffusivity measures across the two meta-ROIs, accounting for specific region weights where applicable.

### 2.5 Statistical analyses

All statistical analyses were performed in R version 2022.07.2 (http://www.r-project.org/). Covariates included age, sex, cognitive status (CU, MCI), race/ethnicity, FSRP scores (excluding points for age), and *APOE*-ε4 carrier status. *APOE*-ε4 carrier status was defined as positive (ε2/ε4, ε3/ ε4, ε4/ ε4) or negative (ε2/ε2, ε2/ε3, ε3/ε3). All predictors were standardized before analyses, and an FDR procedure was used to correct for multiple comparisons. Baseline effects of each of the predictors (hippocampal volume, hippocampal FW, hippocampal FA_FWcorr_, Schwarz AD signature_,_ Schwarz_FW_, Schwarz FA_FWcorr_, McEvoy AD signature, McEvoy_FW_, McEvoy FA_FWcorr_) on cognitive scores were estimated using a general linear model. We also evaluated the interaction between the neuroimaging biomarkers and hippocampal volume on cognitive scores at baseline. Linear mixed-effects regression was used to determine the association between baseline biomarkers and longitudinal cognitive scores. We then analyzed the interaction between longitudinal predictors and aging on cognitive scores. Finally, we conducted a post-hoc competitive model analysis with both cross-sectional and longitudinal data to assess the unique variance each predictor contributed to cognitive function beyond the covariates. We also conducted between-biomarker comparisons to test if there were significant differences between models with the traditional (volumetric) biomarkers, FW biomarkers, and FA_FWcorr_ biomarkers. For the longitudinal competitive model, estimates and p-values were calculated using a variance-covariance matrix and the corresponding R_adj_^2^ values were derived exclusively from the fixed effects. All competitive models were bootstrapped so that the unique variances between each predictor could be statistically compared.

## 3 Results

### 3.1 Participant characteristics

Participants in the current dataset had up to four visits at Baseline (n = 284), 18-months (n = 246), 3-years (n = 215), and 5-years (n = 125). Demographic and clinical variables at baseline for each cognitive status group (CU, MCI) are summarized in Table 1. Participants were mostly well-educated, elderly, non-Hispanic White individuals. There were no significant differences in age, sex distribution, or race/ethnicity distribution between groups. The CU group had more years of education than the MCI group. The MCI group had more *APOE*-ε4 positive individuals than the CU group. As expected, there were significant differences between the CU and MCI groups in all neuropsychological test scores. All neuroimaging biomarkers except hippocampal FA_FWcorr_ also had significant between-group differences (all p < 1.3 x10^-7^).

**Table 1.** Vanderbilt Memory and Aging Project (VMAP) cohort information.

	Total	CU	MCI	p-value
Cohort characteristics				
Number of participants	296	167	129	
Number of sessions	870	529	341	
Longitudinal follow-up (years)	2.8 ± 1.3	2.9 ± 1.4	2.6 ± 1.2	
Number of visits	2.2 ± 1.1	2.3 ± 1.1	2.1 ± 1.0	
Baseline demographics				
Age at baseline (years)	72.7 ± 7.2	72.3 ± 7.0	73.3 ± 7.5	3.33 x 10^-1^
Sex (% female)	41.3	42.5	39.3	3.43 x 10^-1^
Education (years)	16.0 ± 2.6	16.5 ± 2.5	15.2 ± 2.7	**1.80 x 10^-11^**
Race (% Non-Hispanic White)	86.3	86.0	86.8	5.04 x 10^-1^
*APOE*- ε4 (% positive)	36.0	29.3	46.3	**4.85 x 10^-7^**
FSRP (total score)	6.3 ± 3.0	6.1 ± 2.9	6.7 ± 3.1	**2.63 x 10^-3^**
Baseline neuropsychological outcomes				
Montreal cognitive assessment (total score)	24.9 ± 4.0	26.7 ± 2.3	22.2 ± 4.5	
Episodic memory				
Episodic memory composite	0.08 ± 1.0	0.60 ± 0.76	-0.73 ± 0.88	
CVLT-II total learning	41.3 ± 12.9	47.3 ± 10.4	31.9 ± 10.8	
CVLT-II long delay free recall	8.6 ± 4.5	10.7 ± 3.5	5.3 ± 3.9	
CVLT-II recognition	2.5 ± 1.0	3.0 ± 0.8	1.8 ± 1.0	
BFLT total learning	118.0 ± 43.8	139.8 ± 31.4	84.2 ± 38.5	
BFLT long delay free recall	27.7 ± 11.2	33.3 ± 7.5	19.2 ± 10.7	
BFLT recognition	0.74 ± 0.23	0.84 ± 0.14	0.58 ± 0.25	
Executive function				
Executive function composite	-0.01 ± 1.0	0.42 ± 0.66	-0.68 ± 1.07	
DKEFS tower test	16.1 ± 5.0	17.5 ± 4.3	13.9 ± 5.2	
DKEFS letter-number switching*	121.3 ± 105.8	90.3 ± 39.6	172.9 ± 151.6	
DKEFS color-word inhibition^#^	71.0 ± 33.6	60.9 ± 15.2	86.4 ± 46.0	
Letter fluency (FAS)	38.7 ± 11.9	42.5 ± 11.2	32.8 ± 10.5	
Language				
Boston naming test	26.9 ± 3.4	28.0 ± 2.1	25.2 ± 4.3	
Animal naming	18.8 ± 6.0	20.8 ± 5.3	15.7 ± 5.7	
Information processing speed				
WAIS-IV coding	52.6 ± 14.0	57.7 ± 12.1	44.7 ± 13.0	
DKEFS number sequencing^#^	44.9 ± 24.3	37.7 ± 14.1	56.2 ± 31.4	
Visuospatial skills				
Hooper visual organization test	24.5 ± 3.4	25.5 ± 2.4	23.1 ± 4.1	

Significant (p < 0.05) results indicated in bold. p-values for all neuropsychological outcomes < 2.2 x10^-16^.

Abbreviations: *APOE*, apolipoprotein E; BFLT, Biber Figure Learning Test; CVLT-II, California Verbal Learning Test second edition; DKEFS, Delis-Kaplan Executive Function System; FSRP, Framingham Stroke Risk Profile; MCI, mild cognitive impairment; CU, cognitively unimpaired; WAIS-IV, Wechsler Adult Intelligence Scale IV.

# Represents time to completion (s).

* Represents log time to completion (s).

### 3.2 Baseline biomarker association with baseline cognitive performance

**Fig. 2.**
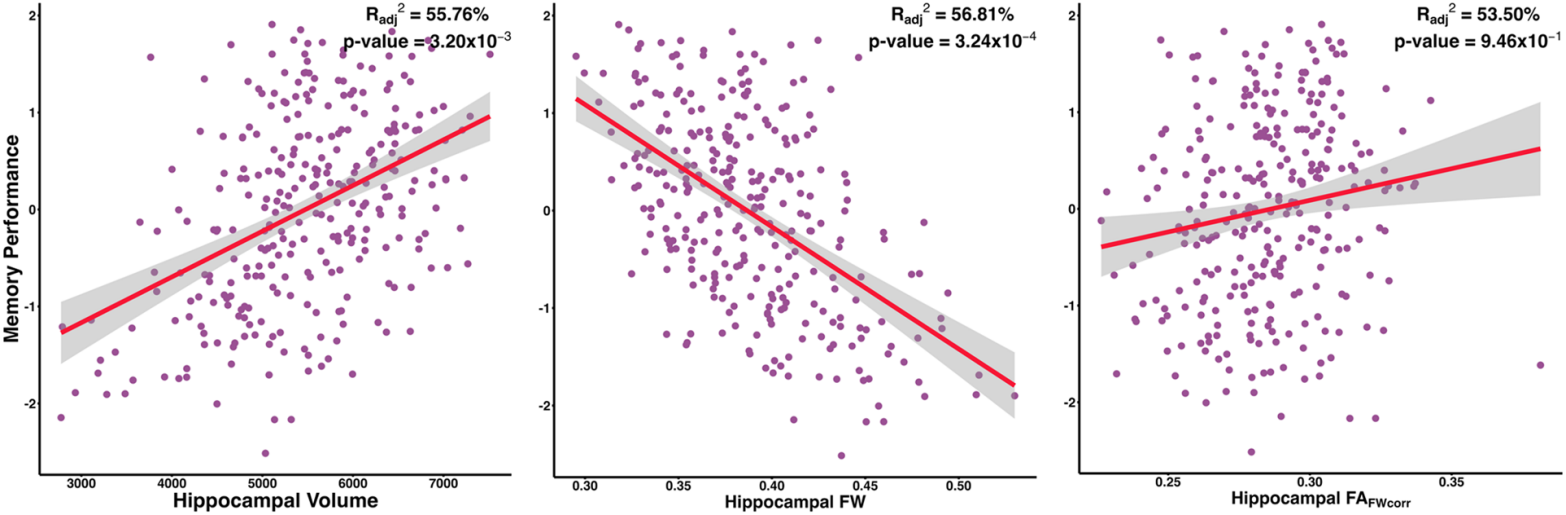
Hippocampal Biomarker Associations with Memory Performance at Baseline.

Equation 1 illustrates the linear regression model used to assess the association between baseline biomarker values and cognitive performance. Baseline results are presented in Table 2. The association of hippocampal measures with memory performance is graphically summarized in Figure 2. Hippocampal volume, hippocampal FW, Schwarz AD Signature, Schwarz_FW_, McEvoy AD Signature, and McEvoy_FW_ all exhibited widespread significant associations with memory performance. Notably, hippocampal FA_FWcorr_ did not exhibit significant associations with any cognitive scores. We did not observe any *predictor × hippocampal volume* interactions on cognitive composites at baseline. Results for this interaction analysis can be found in Supplemental Table 1.

**Table 2.** Neuroimaging biomarker associations with cognitive performance at baseline.

Neuropsychological performance	Hippocampal volume				Hippocampal FW				Hippocampal FA_FWcorr_			
	β	β_SE_	p	p_FDR_	β	β_SE_	p	p_FDR_	β	β_SE_	p	p_FDR_
Episodic memory composite	1.12	0.31	4.09 x 10^-4^	**3.20 x 10^-3^**	-2.13	0.47	9.00 x 10^-6^	**3.24 x 10^-4^**	0.06	0.54	9.07 x 10^-1^	9.46 x 10^-1^
Executive function composite	0.45	0.33	1.73 x 10^-1^	2.83 x 10^-1^	-1.36	0.49	5.94 x 10^-3^	**2.52 x 10^-2^**	1.20	0.55	2.93 x 10^-2^	7.81 x 10^-2^
Boston naming test	2.71	1.26	3.28 x 10^-2^	8.44 x 10^-2^	-3.75	1.92	5.24 x 10^-2^	1.14 x 10^-1^	-1.17	2.15	5.88 x 10^-1^	7.30 x 10^-1^
Animal naming	6.14	2.13	4.21 x 10^-3^	**2.12 x 10^-2^**	-8.65	3.24	8.12 x 10^-3^	**3.08 x 10^-2^**	5.13	3.64	1.60 x 10^-1^	2.71 x 10^-1^
WAIS-IV coding	10.30	4.94	3.78 x 10^-2^	9.09 x 10^-2^	-5.00	7.57	5.09 x 10^-1^	6.55 x 10^-1^	13.67	8.39	1.04 x 10^-1^	1.92 x 10^-1^
DKEFS number sequencing	1.30	8.40	8.77 x 10^-1^	9.29 x 10^-1^	12.39	12.76	3.32 x 10^-1^	4.72 x 10^-1^	-25.01	14.14	7.79 x 10^-2^	1.56 x 10^-1^
Hooper visual organization test	3.63	1.31	5.92 x 10^-3^	**1.18 x 10^-2^**	-5.61	1.99	5.12 x 10^-3^	**1.18 x 10^-2^**	1.65	2.24	4.63 x 10^-1^	4.63 x 10^-1^

	Schwarz AD signature				Schwarz FW				Schwarz FA_FWcorr_			
	β	β_SE_	p	p_FDR_	β	β_SE_		p_FDR_	β	β_SE_		p_FDR_
Episodic memory composite	2.85	0.72	8.81 x 10^-5^	**1.22 x 10^-3^**	-1.36	0.42	1.58 x 10^-3^	**1.04 x 10^-2^**	-1.76	0.74	1.77 x 10^-2^	5.53 x 10^-2^
Executive function composite	1.76	0.75	1.89 x 10^-2^	5.66 x 10^-2^	-1.54	0.43	4.44 x 10^-4^	**3.20 x 10^-3^**	-1.48	0.76	5.15 x 10^-2^	1.14 x 10^-1^
Boston naming test	11.16	2.85	1.11 x 10^-4^	**1.22 x 10^-3^**	-3.55	1.71	3.82 x 10^-2^	9.09 x 10^-2^	-7.74	2.93	8.65 x 10^-3^	**3.11 x 10^-2^**
Animal naming	6.41	4.94	1.96 x 10^-1^	3.00 x 10^-1^	-10.31	2.85	3.50 x 10^-4^	**3.15 x 10^-3^**	-12.88	4.97	1.00 x 10^-2^	**3.44 x 10^-2^**
WAIS-IV coding	9.09	11.42	4.27 x 10^-1^	5.80 x 10^-1^	-8.90	6.70	1.85 x 10^-1^	2.90 x 10^-1^	-16.54	11.56	1.54 x 10^-1^	2.71 x 10^-1^
DKEFS number sequencing	-0.38	19.29	9.84 x 10^-1^	9.84 x 10^-1^	19.13	11.28	9.11 x 10^-2^	1.77 x 10^-1^	26.37	19.50	1.77 x 10^-1^	2.84 x 10^-1^
Hooper visual organization test	5.97	3.02	4.93 x 10^-2^	7.40 x 10^-2^	-4.59	1.77	1.00 x 10^-2^	**1.71 x 10^-2^**	-11.73	3.01	1.21 x 10^-4^	**1.45 x 10^-3^**

	McEvoy AD signature				McEvoy FW				McEvoy FA_FWcorr_			
	β	β_SE_	p	p_FDR_	β	β_SE_	p	p_FDR_	β	β_SE_	p	p_FDR_
Episodic memory composite	0.21	0.04	2.52 x 10^-6^	**1.81 x 10^-4^**	-1.92	0.45	**2.66 x 10^-5^**	6.37 x 10^-4^	-1.94	0.85	2.41 x 10^-2^	6.95 x 10^-2^
Executive function composite	0.14	0.05	2.94 x 10^-3^	**1.63 x 10^-2^**	-1.47	0.47	**1.74 x 10^-3^**	1.05 x 10^-2^	-1.43	0.88	1.04 x 10^-1^	1.92 x 10^-1^
Boston naming test	0.72	0.18	6.42 x 10^-5^	**1.15 x 10^-3^**	-5.25	1.81	**4.09 x 10^-3^**	2.01 x 10^-2^	-9.67	3.38	4.47 x 10^-3^	**2.01 x 10^-2^**
Animal naming	0.78	0.30	1.07 x 10^-2^	**3.50 x 10^-2^**	-11.87	3.04	**1.19 x 10^-4^**	1.22 x 10^-3^	-12.69	5.76	2.83 x 10^-2^	7.81 x 10^-2^
WAIS-IV coding	0.86	0.71	2.27 x 10^-1^	3.33 x 10^-1^	-8.83	7.18	2.20 x 10^-1^	3.30 x 10^-1^	-12.76	13.37	3.41 x 10^-1^	4.72 x 10^-1^
DKEFS number sequencing	-0.24	1.20	8.42 x 10^-1^	9.29 x 10^-1^	16.99	12.11	1.62 x 10^-1^	2.71 x 10^-1^	17.07	22.57	4.50 x 10^-1^	6.00 x 10^-1^
Hooper visual organization test	0.53	0.19	4.94 x 10^-3^	**1.18 x 10^-2^**	-5.34	1.89	**5.10 x 10^-3^**	1.18 x 10^-2^	-11.92	3.50	7.45 x 10^-4^	**4.47 x 10^-3^**

Significant (p_FDR_< 0.05) results indicated in bold. Abbreviations: FWcorr, free-water corrected; FA, fractional anisotropy.

$$\begin{array}{l} Cognitive.score\sim Biomarker+age+sex+education\\ \;\;\;\;\;+race+APOE4.positivity+diagnosis+fsrp.score \end{array}$$ (Equation 1)

### 3.3 Baseline competitive model analysis

We then conducted a competitive model analysis to determine the unique variance explained by hippocampal volume and other neuroimaging biomarkers in addition to all covariates (age, sex, education, cognitive status, *APOE*-ε4 status, race, FSRP scores). All predictors were standardized, and measures for the adjusted R-squared (R_adj_^2^) value and its standard deviation were bootstrapped for robustness. We found that covariates explained approximately 54% of variance in memory performance (R_adj_^2^ = 53.74 ± 3.84%). We then iteratively added structural and diffusivity metrics to this model. Results for each predictor can be found in Table 3. The R_adj_^2^ column represents the adjusted R-squared value ± standard deviation derived from Equation 1. The ΔR^2^_adj_ column represents the difference between adjusted R-squared values from this model and the covariates-only model (imaging biomarkers not included as predictors) and its standard deviation.

**Table 3.** Baseline competitive model analysis to determine the unique variance explained by biomarkers beyond covariates.

Neuropsychological performance	Hippocampal volume		Hippocampal FW		Hippocampal FA_FWcorr_	
	R^2^_adj_	ΔR^2^_adj_	R^2^_adj_	ΔR^2^_adj_	R^2^_adj_	ΔR^2^_adj_
Episodic memory composite	**55.76 ± 3.79^bc^**	**1.96 ± 1.10**	**56.81 ± 3.42^ac^**	**3.07 ± 1.54**	53.50 ± 3.92^ab^	0 ± 0.22
Executive function composite	45.86 ± 4.03^bc^	0.38 ± 0.63	**47.12 ± 4.00^ac^**	**1.51 ± 1.07**	**46.57 ± 4.09^ab^**	**0.91 ± 0.87**
Boston naming test	**29.83 ± 4.54^c^**	**1.20 ± 1.25**	**29.44 ± 4.49**	**0.95 ± 1.15**	28.91 ± 4.32^a^	0.12 ± 0.49
Animal naming	**34.14 ± 4.65^c^**	**1.87 ± 1.30**	**34.18 ± 4.52^c^**	**1.57 ± 1.19**	33.00 ± 4.57^ab^	0.39 ± 0.61
WAIS-IV coding	**35.93 ± 4.68**	**0.92 ± 0.94**	35.43 ± 4.48	0.07 ± 0.39	**35.65 ± 4.60**	**0.64 ± 0.85**
DKEFS number sequencing	26.47 ± 4.48^c^	0.10 ± 0.50	26.49 ± 4.53^c^	0.15 ± 0.48	**27.18 ± 4.68^ab^**	**0.75 ± 0.81**
Hooper visual organization	**27.34 ± 4.32^c^**	**1.94 ± 1.48**	**27.22 ± 4.31^c^**	**2.00 ± 1.52**	25.57 ± 4.50^ab^	0.24 ± 0.67

	Schwarz AD signature			Schwarz FW	Schwarz FA_FWcorr_	
	R^2^_adj_	ΔR^2^_adj_	R^2^_adj_	ΔR^2^_adj_	R^2^_adj_	ΔR^2^_adj_
Episodic memory composite	**55.90 ± 3.71^bc^**	**2.44 ± 1.41**	**55.14 ± 3.74^a^**	**1.62 ± 1.08**	**54.67 ± 3.60^a^**	**0.88 ± 0.73**
Executive function composite	**46.54 ± 3.82^b^**	**0.98 ± 0.99**	**47.93 ± 3.93^ac^**	**2.43 ± 1.63**	**46.49 ± 3.82^b^**	**0.71 ± 0.76**
Boston naming test	**32.27 ± 4.77^bc^**	**3.62 ± 2.01**	**30.05 ± 4.67^a^**	**1.30 ± 1.49**	**30.21 ± 4.38^a^**	**1.74 ± 1.43**
Animal naming	33.04 ± 4.57^bc^	0.38 ± 0.70	**35.44 ± 4.38^ac^**	**3.00 ± 1.90**	**34.14 ± 4.52^ab^**	**1.57 ± 1.22**
WAIS-IV coding	35.38 ± 4.73	0.21 ± 0.56	35.45 ± 4.59	0.50 ± 0.81	35.49 ± 4.67	0.44 ± 0.66
DKEFS number sequencing	26.29 ± 4.67^b^	-0.02 ± 0.33	**27.22 ± 4.65^a^**	**0.97 ± 1.26**	26.79 ± 4.61	0.54 ± 0.90
Hooper visual organization	**25.91 ± 4.02^bc^**	**1.00 ± 1.12**	**26.86 ± 4.22^ac^**	**1.76 ± 1.55**	**29.05 ± 4.19^ab^**	**3.79 ± 1.95**

	McEvoy AD signature			McEvoy FW	McEvoy FA_FWcorr_	
	R^2^_adj_	ΔR^2^_adj_	R^2^_adj_	ΔR^2^_adj_	R^2^_adj_	ΔR^2^_adj_
Episodic memory composite	**57.16 ± 3.68^bc^**	**3.47 ± 1.65**	**56.49 ± 3.80^ac^**	**2.74 ± 1.38**	**54.56 ± 3.76^ab^**	**0.79 ± 0.67**
Executive function composite	**47.51 ± 3.86^c^**	**1.77 ± 1.42**	**47.49 ± 4.13^c^**	**1.90 ± 1.45**	**46.21 ± 4.01^ab^**	**0.52 ± 0.73**
Boston naming test	**32.52 ± 4.93^bc^**	**3.89 ± 2.11**	**30.45 ± 4.80^a^**	**2.22 ± 1.97**	**30.57 ± 4.39^a^**	**1.99 ± 1.45**
Animal naming	**34.11 ± 4.61^b^**	**1.62 ± 1.45**	**36.15 ± 4.35^ac^**	**3.57 ± 2.05**	**33.67 ± 4.34^b^**	**1.11 ± 1.03**
WAIS-IV coding	35.74 ± 4.45	0.34 ± 0.67	35.58 ± 4.68	0.35 ± 0.66	35.30 ± 4.44	0.22 ± 0.56
DKEFS number sequencing	26.20 ± 4.64^b^	0.01 ± 0.37	**26.98 ± 4.69^a^**	**0.61 ± 1.01**	26.56 ± 4.69	0.27 ± 0.78
Hooper visual organization	**27.22 ± 4.08^c^**	**1.94 ± 1.49**	**27.20 ± 4.27^c^**	**2.15 ± 1.79**	**28.44 ± 4.42^ab^**	**2.93 ± 1.88**

Measures that are significantly different from the covariates-only model are indicated in bold. Superscript indicates that the measure was significantly different from the (a) traditional model, (b) FW model, or (c) FA_FWcorr_ model.

Abbreviations: FWcorr, free-water corrected; FA, fractional anisotropy.

For the hippocampal models, we found that hippocampal FW was the superior metric in the association with memory composite performance and executive function performance. Hippocampal volume and FW performed similarly for the Boston Naming Test and Animal Naming. While hippocampal volume and hippocampal FA_FWcorr_ outperformed the covariate model for WAIS-IV Coding, there were no significant differences between the biomarkers. For DKEFS Number Sequencing, hippocampal FA_FWcorr_ was superior to hippocampal volume and FW. For the Hooper Visual Organization Test, hippocampal volume and FW performed similarly and were both superior to hippocampal FA_FWcorr_. Table 3 illustrates all between-biomarker comparisons for each cognitive test.

### 3.4 Baseline biomarker association with longitudinal cognitive performance

**Fig. 3.**
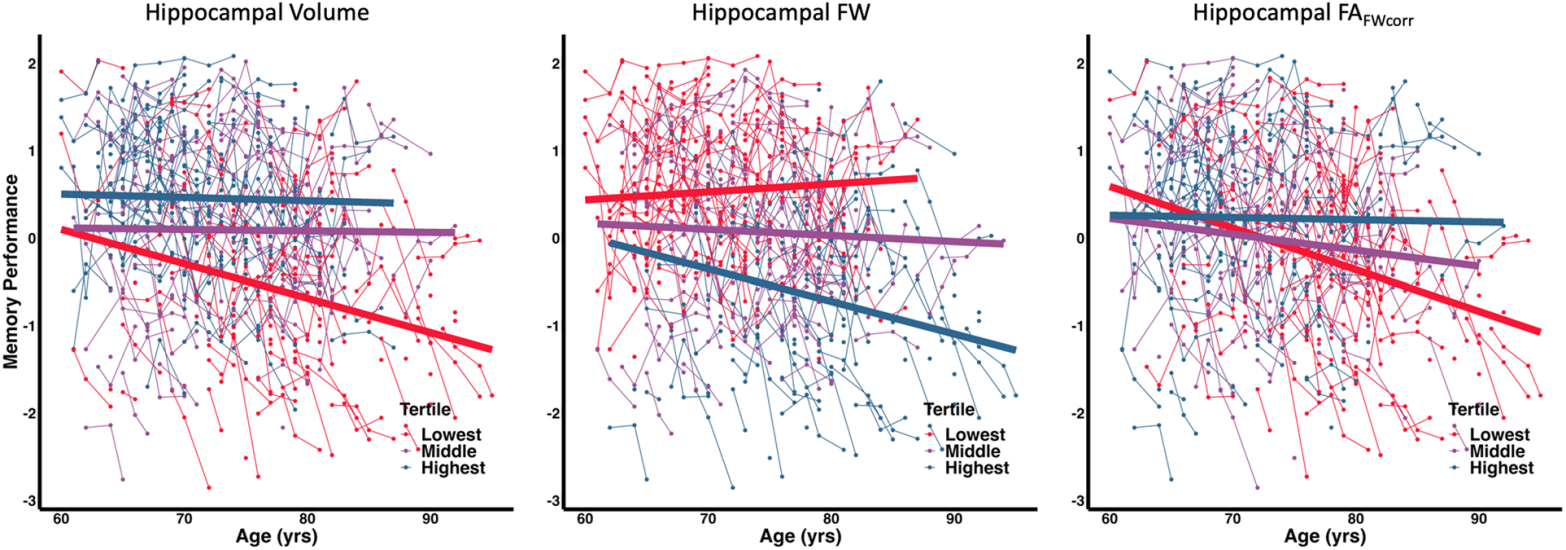
Baseline Hippocampal Biomarker Associations with Longitudinal Memory Performance. Each biomarker is colored by the lowest (red), middle (purple), and highest (blue) tertile.

Equation 2 illustrates the linear mixed-effects regression model used to assess the association between baseline biomarker values and longitudinal cognitive performance. All predictors were standardized, and an FDR procedure was used to correct for multiple comparisons. Longitudinal results are presented in Table 4. Boxplots showing the mean and standard deviation of FW measures and cognitive test scores at each visit are available in Supplemental Figures 1 and 2 respectively. We found that Hippocampal FW had the lowest FDR-corrected p-values as a predictor for both episodic memory and executive function composite scores. The association of baseline hippocampal measures with longitudinal memory performance is graphically summarized in Figure 3. Splitting the data into tertiles reveals that individuals with the lowest hippocampal volume and hippocampal FA_FWcorr_, and the highest hippocampal FW had the greatest rate of decline in memory performance with age.

**Table 4.** Baseline biomarker associations with longitudinal cognitive performance.

Neuropsychological performance	Hippocampal volume				Hippocampal FW				Hippocampal FA_FWcorr_			
	β	β_SE_	p	p_FDR_	β	β_SE_	p	p_FDR_	β	β_SE_	p	p_FDR_
Episodic memory composite	1.49	0.30	5.68 x 10^-7^	**5.86 x 10^-6^**	-3.03	0.41	2.71 x 10^-13^	**1.95 x 10^-11^**	0.49	0.53	3.50 x 10^-1^	4.35 x 10^-1^
Executive function composite	0.68	0.31	2.92 x 10^-2^	5.25 x 10^-2^	-1.96	0.44	8.49 x 10^-6^	**5.09 x 10^-5^**	0.93	0.54	8.65 x 10^-2^	1.35 x 10^-1^
Boston naming test	3.28	1.18	5.54 x 10^-3^	**1.37 x 10^-2^**	-4.99	1.71	3.58 x 10^-3^	**9.21 x 10^-3^**	-1.27	2.07	5.39 x 10^-1^	6.19 x 10^-1^
Animal naming	7.34	1.93	1.46 x 10^-4^	**6.59 x 10^-4^**	-13.94	2.76	4.43 x 10^-7^	**5.86 x 10^-6^**	8.77	3.32	8.26 x 10^-3^	**1.98 x 10^-2^**
WAIS-IV coding	11.76	4.68	1.19 x 10^-2^	**2.61 x 10^-2^**	-12.19	6.79	7.27 x 10^-2^	1.22 x 10^-1^	16.30	8.06	4.32 x 10^-2^	7.58 x 10^-2^
DKEFS number sequencing	-4.31	7.07	5.42 x 10^-1^	6.19 x 10^-1^	23.32	10.23	2.26 x 10^-2^	**4.28 x 10^-2^**	-20.67	11.90	8.24 x 10^-2^	1.32 x 10^-1^
Hooper visual organization test	4.74	1.24	1.39 x 10^-4^	**3.64 x 10^-4^**	-8.24	1.79	3.93 x 10^-6^	**4.72 x 10^-5^**	2.92	2.17	1.79 x 10^-1^	2.14 x 10^-1^

	Schwarz AD signature				Schwarz FW				Schwarz FA_FWcorr_			
	β	β_SE_	p	p_FDR_	β	β_SE_	p	p_FDR_	β	β_SE_	p	p_FDR_
Episodic memory composite	4.07	0.68	1.96 x 10^-9^	**4.21 x 10^-8^**	-1.91	0.39	1.21 x 10^-6^	**9.64 x 10^-6^**	-2.14	0.72	2.95 x 10^-3^	**8.15 x 10^-3^**
Executive function composite	2.25	0.71	1.60 x 10^-3^	**5.47 x 10^-3^**	-1.59	0.41	1.15 x 10^-4^	**5.51 x 10^-4^**	-1.93	0.74	9.32 x 10^-3^	**2.17 x 10^-2^**
Boston naming test	13.19	2.69	9.73 x 10^-7^	**8.76 x 10^-6^**	-3.92	1.56	1.19 x 10^-2^	**2.61 x 10^-2^**	-8.35	2.75	2.39 x 10^-3^	**7.48 x 10^-3^**
Animal naming	16.21	4.50	3.21 x 10^-4^	**1.28 x 10^-3^**	-11.22	2.55	1.08 x 10^-5^	**5.97 x 10^-5^**	-10.54	4.61	2.23 x 10^-2^	**4.28 x 10^-2^**
WAIS-IV coding	11.33	11.05	3.05 x 10^-1^	3.85 x 10^-1^	-10.35	6.15	9.24 x 10^-2^	1.42 x 10^-1^	-12.02	11.26	2.85 x 10^-1^	3.67 x 10^-1^
DKEFS number sequencing	-17.62	16.25	2.78 x 10^-1^	3.64 x 10^-1^	28.22	9.53	3.06 x 10^-3^	**8.15 x 10^-3^**	28.81	16.16	7.47 x 10^-2^	1.22 x 10^-1^
Hooper visual organization test	10.07	2.91	5.32 x 10^-4^	**7.98 x 10^-4^**	-6.22	1.64	1.54 x 10^-4^	**3.64 x 10^-4^**	-12.24	2.90	1.89 x 10^-5^	**1.14 x 10^-4^**

	McEvoy AD signature				McEvoy FW				McEvoy FA_FWcorr_			
	β	β_SE_	p	p_FDR_	β	β_SE_	p	p_FDR_	β	β_SE_	p	p_FDR_
Episodic memory composite	0.24	0.04	7.23 x 10^-10^	**2.60 x 10^-8^**	-2.45	0.41	2.34 x 10^-9^	**4.21 x 10^-8^**	-2.75	0.83	9.76 x 10^-4^	**3.51 x 10^-3^**
Executive function composite	0.16	0.04	1.11 x 10^-4^	**5.51 x 10^-4^**	-1.57	0.44	3.03 x 10^-4^	**1.28 x 10^-3^**	-2.00	0.86	2.02 x 10^-2^	**4.13 x 10^-2^**
Boston naming test	0.75	0.16	2.09 x 10^-6^	**1.51 x 10^-5^**	-4.95	1.64	2.62 x 10^-3^	**7.53 x 10^-3^**	-9.75	3.21	2.37 x 10^-3^	**7.48 x 10^-3^**
Animal naming	1.18	0.26	6.10 x 10^-6^	**3.99 x 10^-5^**	-13.42	2.68	5.69 x 10^-7^	**5.86 x 10^-6^**	-10.10	5.37	6.01 x 10^-2^	1.03 x 10^-1^
WAIS-IV coding	0.91	0.65	1.63 x 10^-1^	2.35 x 10^-1^	-10.01	6.50	1.23 x 10^-1^	1.81 x 10^-1^	-8.99	13.04	4.91 x 10^-1^	5.89 x 10^-1^
DKEFS number sequencing	-1.20	0.94	2.00 x 10^-1^	2.77 x 10^-1^	23.49	10.15	2.07 x 10^-2^	**4.13 x 10^-2^**	21.06	18.97	2.67 x 10^-1^	3.56 x 10^-1^
Hooper visual organization test	0.63	0.17	2.12 x 10^-4^	**3.64 x 10^-4^**	-6.58	1.74	1.51 x 10^-4^	**3.64 x 10^-4^**	-12.66	3.40	1.97 x 10^-4^	**3.64 x 10^-4^**

Significant (p_FDR_< 0.05) results indicated in bold.

Abbreviations: FWcorr, free-water corrected; FA, fractional anisotropy.

$$\begin{array}{l} Cognitive.score\sim Biomarker+age+sex+education\\ \;\;\;\;\;+race+APOE4.positivity+diagnosis.baseline\\ \;\;\;\;\;+fsrp.score+(1+age|Participant.ID) \end{array}$$ (Equation 2)

Additionally, a linear mixed-effects analysis was conducted to examine the association between baseline diagnostic status and longitudinal biomarker values. Results for this analysis can be found in Supplemental Table 2. We found that the McEvoy AD Signature was the only biomarker among the nine considered that did not show a significant association with baseline diagnostic status.

We also evaluated *predictor × age* and *predictor × diagnosis.baseline* interactions on longitudinal cognitive scores. Results for these analyses can be found in Supplemental Tables 3 and 4, respectively. Most neuroimaging biomarkers showed significant interaction with aging, with *hippocampal FW × age* interactions producing the lowest FDR-corrected p-values for most cognitive scores.

### 3.5 Longitudinal competitive model analysis

We also conducted a competitive model analysis to determine the unique marginal variance explained by neuroimaging biomarkers, in addition to all covariates (age, sex, education, cognitive status, *APOE*-ε4 status, race, FSRP scores). All predictors were standardized, and measures for the R_adj_^2^ value and its standard deviation were bootstrapped for robustness. Difference maps illustrating the differences in FW and FA_FWcorr_ values between CU and MCI groups at each study timepoint can be found in Supplemental Figures 3 and 4 respectively. Results indicate that covariates explained approximately 39% of variance in memory performance (R_adj_^2^ = 39.29 ± 3.22 %). We then iteratively added structural and diffusivity metrics to this model. Results for each predictor can be found in Table 5. The R_adj_^2^ column represents the adjusted R-squared value ± standard deviation derived from Equation 2. The ΔR^2^_adj_ column represents the difference between adjusted R-squared values from this model and the covariates-only model (imaging biomarkers not included as predictors) and its standard deviation.

**Table 5.** Longitudinal competitive model analysis to determine the unique variance explained by biomarkers beyond covariates.

Neuropsychological performance	Hippocampal volume		Hippocampal FW		Hippocampal FA_FWcorr_	
	R^2^_adj_	ΔR^2^_adj_	R^2^_adj_	ΔR^2^_adj_	R^2^_adj_	ΔR^2^_adj_
Episodic memory composite	**43.33 ± 3.17^bc^**	**4.02 ± 0.84**	**47.48 ± 3.26^ac^**	**8.13 ± 1.25**	39.24 ± 3.18^ab^	0.03 ± 0.21
Executive function composite	**34.73 ± 4.91^b^**	**0.95 ± 0.72**	**37.53 ± 5.13^ac^**	**3.83 ± 1.24**	34.44 ± 4.76^b^	0.50 ± 0.49
Boston naming test	**17.39 ± 3.80^bc^**	**2.27 ± 0.95**	**18.29 ± 3.79^ac^**	**3.06 ± 1.30**	15.02 ± 3.26^ab^	-0.16 ± 0.20
Animal naming	**30.40 ± 2.93^bc^**	**3.25 ± 0.95**	**32.70 ± 2.96^ac^**	**5.57 ± 1.55**	**28.11 ± 3.07^ab^**	**1.10 ± 0.57**
WAIS-IV coding	**28.63 ± 4.15^c^**	**1.61 ± 0.82**	**28.32 ± 4.01**	**1.01 ± 0.71**	**27.83 ± 4.14^a^**	**0.68 ± 0.56**
DKEFS number sequencing	22.21 ± 3.58^bc^	0.07 ± 0.25	**23.28 ± 3.70^a^**	**0.80 ± 0.54**	**23.16 ± 3.83^a^**	**0.60 ± 0.94**
Hooper visual organization	**15.90 ± 3.54^bc^**	**3.06 ± 1.16**	**17.70 ± 3.88^ac^**	**4.61 ± 0.14**	**13.27 ± 3.21^ab^**	**0.43 ± 0.46**

	Schwarz AD signature		Schwarz FW		Schwarz FA_FWcorr_	
	R^2^_adj_	ΔR^2^_adj_	R^2^_adj_	ΔR^2^_adj_	R^2^_adj_	ΔR^2^_adj_
Episodic memory composite	**45.06 ± 3.28^bc^**	**5.50 ± 1.00**	**43.27 ± 3.24^ac^**	**3.88 ± 0.94**	**41.08 ± 3.26^ab^**	**1.65 ± 0.49**
Executive function composite	**36.03 ± 4.72^bc^**	**1.90 ± 0.81**	**37.17 ± 4.59^ac^**	**2.92 ± 1.07**	**35.04 ± 4.69^ab^**	**1.03 ± 0.56**
Boston naming test	**19.68 ± 4.04^bc^**	**4.57 ± 1.50**	**17.27 ± 3.67^a^**	**1.94 ± 1.10**	**16.83 ± 3.77^a^**	**1.73 ± 0.91**
Animal naming	**29.71 ± 2.81^bc^**	**2.70 ± 0.87**	**31.19 ± 3.18^ac^**	**4.10 ± 1.14**	**28.18 ± 3.15^ab^**	**1.11 ± 1.07**
WAIS-IV coding	27.21 ± 3.93^b^	0.30 ± 0.37	**27.91 ± 4.10^ac^**	**0.79 ± 0.65**	27.33 ± 3.99^b^	0.21 ± 0.29
DKEFS number sequencing	22.72 ± 3.30^b^	0.23 ± 0.31	**24.03 ± 3.89^ac^**	**1.53 ± 1.47**	**23.10 ± 3.67^b^**	**0.68 ± 1.16**
Hooper visual organization	**15.24 ± 3.50^c^**	**2.15 ± 0.93**	**15.58 ± 3.56**	**2.63 ± 1.12**	**15.72 ± 3.64^a^**	**2.84 ± 1.22**

	McEvoy AD signature		McEvoy FW		McEvoy FA_FWcorr_	
	R^2^_adj_	ΔR^2^_adj_	R^2^_adj_	ΔR^2^_adj_	R^2^_adj_	ΔR^2^_adj_
Episodic memory composite	**44.31 ± 3.34^bc^**	**4.93 ± 0.95**	**44.75 ± 3.25^ac^**	**5.46 ± 1.07**	**41.34 ± 3.31^ab^**	**2.02 ± 0.56**
Executive function composite	**36.12 ± 4.97^c^**	**2.00 ± 0.89**	**36.57 ± 4.98^c^**	**2.48 ± 1.10**	**34.91 ± 4.66^ab^**	**0.79 ± 0.70**
Boston naming test	**18.60 ± 4.11^bc^**	**3.49 ± 1.29**	**17.82 ± 3.95^ac^**	**2.67 ± 1.27**	**16.90 ± 3.93^ab^**	**1.70 ± 0.95**
Animal naming	**30.51 ± 3.01^bc^**	**3.45 ± 1.04**	**32.14 ± 2.85^ac^**	**5.04 ± 1.37**	**27.89 ± 2.88^ab^**	**0.77 ± 0.45**
WAIS-IV coding	27.43 ± 3.93	0.19 ± 0.27	**27.82 ± 4.08^c^**	**0.74 ± 0.67**	26.93 ± 3.89^b^	-0.06 ± 0.22
DKEFS number sequencing	22.80 ± 3.25^b^	0.18 ± 0.74	**23.49 ± 3.72^ac^**	**0.93 ± 0.78**	22.81 ± 3.45^b^	0.31 ± 0.74
Hooper visual organization	**14.40 ± 3.41^bc^**	**1.59 ± 0.86**	**15.87 ± 3.55^ac^**	**2.88 ± 1.20**	**15.41 ± 3.62^ab^**	**2.37 ± 1.00**

Measures that are significantly different from the covariates-only model are indicated in bold. Superscript indicates that the measure was significantly different from the (a) traditional model, (b) FW model, or (c) FA_FWcorr_ model.

Abbreviations: FWcorr, free-water corrected; FA, fractional anisotropy.

For the hippocampal models, hippocampal FW was the superior biomarker for the memory composite, executive function composite, Boston Naming Test, Animal Naming Test, and Hooper Visual Organization Test. For WAIS-IV Coding, hippocampal volume outperformed hippocampal FA_FWcorr_, but did not outperform hippocampal FW. For DKEFS Number Sequencing, hippocampal FW and FA_FWcorr_ both outperformed hippocampal volume. For the Schwarz and McEvoy models, the FW biomarker was superior across most cognitive tests. Table 5 illustrates all between-biomarker comparisons for each cognitive test.

## 4 Discussion

The current study leveraged cross-sectional neuroimaging and longitudinal cognitive data to investigate the relationship of structural and dMRI biomarkers with cognitive performance. Structural MRI biomarkers included hippocampal volume, McEvoy AD Signature, and Schwarz AD Signature; dMRI biomarkers included FW-corrected metrics (FW, FA_FWcorr_) sampled from the same ROIs as the structural biomarkers. We conducted linear regression analyses to determine the associations between each biomarker and cross-sectional cognitive performance. We then conducted linear mixed-effects regression analyses to determine the associations between baseline biomarkers and longitudinal cognitive performance. Next, we conducted competitive model analyses to determine the unique variance explained by each biomarker beyond covariates for both baseline and longitudinal data. We report three primary findings. First, we found that hippocampal FW and FW within the Schwarz and McEvoy meta-ROIs was significantly associated with cross-sectional memory and executive function. Second, we found that hippocampal FW was the most statistically significant predictor of longitudinal cognitive decline. Lastly, we found that the incorporation of FW metrics led to a significant increase in the variance explained for cognition beyond covariates. Thus, the current study suggests that FW is a strong predictor of both cognitive impairment and decline and may provide greater sensitivity to microstructural changes compared to traditional measures.

Hippocampal involvement in AD disease progression has been well established by previous literature (Braak et al., 2008; Coupé et al., 2019), leading to the identification of hippocampal volume as a principal biomarker in monitoring AD progression (Jack et al., 2018). While hippocampal atrophy also accompanies healthy aging (Jack et al., 1998), it occurs at an accelerated rate in AD patients as compared to controls and is observed in AD patients prior to the onset of clinical symptoms (Fox et al., 1996). Moreover, hippocampal volume has been associated with impairment in various cognitive functions, including verbal (Fox et al., 1996) and visual episodic memory (Zammit et al., 2017). While hippocampal volume has been extensively explored as a structural AD biomarker, the use of dMRI provides additional sensitivity towards microstructural changes. New methods for modeling complex diffusion, such as FW correction, can provide early and measurable biomarkers of AD pathology (Walsh et al., 2020). Studies have consistently shown that elevated diffusivity and lower FA_CONV_, indicative of more isotropic motion, are consistent with neurodegenerative changes and can detect early-stage changes of AD. Our cross-sectional findings extend previous work, indicating that the hippocampal FW index is a more significant predictor of cognitive performance than hippocampal volume. Ofori et al. (2019) also found that hippocampal FW values were associated with global PET AV45 and CSF Aβ_1–42_ levels and not CSF p-tau and CSF t-tau, suggesting that the FW index may provide specific information about the earliest pathological process in AD.

We found that FW within the Schwarz and McEvoy signature outperformed their traditional measures in most cognitive domains. These findings support the hypothesis that increased levels of FW in the hippocampus and associated ROIs have functional and clinical relevance in the AD process and may be among the earliest detectable structural changes (Hoy et al., 2017). FW is suggested to estimate the size of the extracellular space and reflect the degenerative processes in the area. An increase in the FW index may be associated with inflammatory reactions or neuronal atrophy (Hoy et al., 2017; Ji et al., 2017; Ofori et al., 2019), which result in potentially larger amounts of extracellular fluid. In the current study, hippocampal FW outperformed all other measures examined to predict longitudinal memory and executive function performance. Notably, FW within the Schwarz and McEvoy AD signature are the most significant predictors for most models, even though these signatures are tailored towards quantifying cortical thinning and volumetric loss; thus, a more customized signature incorporating both FW and FA_FWcorr_ may provide even more enhanced associations with cognitive impairment and decline. Notably, we found several negative associations with Schwarz and McEvoy FA_FWcorr_ and cognition, particularly for longitudinal cognitive decline (see Table 4). This contradicts the idea that higher FA is associated with better tissue health and cognitive function. However, two caveats exist in this study. Primarily, we addressed partial volume limitations in the FA measure by conducting FW correction. Prior studies showing large positive associations with FA and cognition may be diminished following FW correction. Secondarily, we examined FA_FWcorr_ within the gray matter, which has a more complex architecture than white matter. Higher complexity in the gray matter may be indicative of healthier tissue and would link lower FA_FWcorr_ with better cognitive performance and less cognitive decline. Future large-scale studies and genetic research may clarify these associations and underlying biological processes.

The present study has several strengths. First, we evaluated several well-established neuroimaging biomarkers which are widely used in the AD field, including hippocampal volume, the Schwarz AD Signature, and the McEvoy AD Signature. In addition to quantifying their traditional values, we also quantified conventional and FW-corrected dMRI measures within these meta-ROIs. Second, we used a longitudinal cohort which has comprehensive neuropsychological assessments and is enriched for MCI—this allowed us to determine how baseline neuroimaging measures in early-stage disease were related to future cognitive decline. While we incorporated several neuroimaging markers in addition to a comprehensive longitudinal cohort, this study is not without its limitations. For example, the VMAP cohort includes well-educated, mostly non-Hispanic white, participants; incorporating cohorts with more diverse backgrounds is necessary. Additionally, although the VMAP cohort is large (n = 296 in the present study), multi-cohort studies may facilitate more statistically robust results. Finally, we used a FW correction technique on single-shell dMRI data. Even though this technique allowed us to conduct more sensitive analyses, the incorporation of multi-shell dMRI acquisitions would allow for more advanced multi-compartment analyses (H. Zhang et al., 2012).

In summary, our findings support FW as a distinct and sensitive in-vivo biomarker for aging and MCI, which could provide unique insight into AD disease progression aside from volumetric measures. Increased FW in the hippocampus and other AD-vulnerable ROIs may reflect a pathophysiological process along the AD continuum, prior to overt evidence of neurodegeneration in the form of atrophy. Ongoing analysis is investigating these findings to determine the cortical and subcortical regions showing the earliest changes in diffusivity. Current research within our team is also exploring the involvement and significance of specific white matter tracts—in tandem with gray matter changes—in age-related cognitive decline. Further work is needed to examine changes in global FW values and association with AD in a multi-ethnic cohort. Finally, as early biomarkers are of increasing interest, future studies should also include cohorts distinguishing between mild/moderate/severe MCI to understand the utility of an FW-DTI in screening for and/or diagnosing AD across all stages of the disease.

## Data and Code Availability

Data from the VMAP cohort can be accessed freely following data use approval (www.vmacdata.org). The source code for the analysis conducted in this study is available on request.

## Author Contributions

A.S. implemented the project; analyzed the results; created the figures; and wrote the first draft of the manuscript. Y.Y. assisted in experimental design and results interpretation. N.S., E.M., and K.R.P. assisted in data gathering and results interpretation. L.D., K.A.G., T.J.H., and A.L.J. obtained funding and resources used for the project and provided guidance in data use and experimental design. K.G.S. and B.A.L. provided guidance in experimental design and results interpretation. D.B.A. obtained funding and resources used for the project and was primary supervisor in all aspects. All authors contributed to revisions of the manuscript prior to submission.

## Funding

This study was supported by several funding sources, including K01EB032898 (K.G.S.), K01AG073584 (D.B.A.), U24AG074855 (T.J.H.), 75N95D22P00141 (T.J.H.), and R01AG059716 (T.J.H.). The research was support in part by the Intramural Research Program of the National Institutes of Health, National Institute on Aging. Study data were obtained from the Vanderbilt Memory and Aging Project (VMAP). VMAP data were collected by Vanderbilt Memory and Alzheimer’s Center Investigators at Vanderbilt University Medical Center. This work was supported by NIA grants R01AG034962 (PI: A.L.J.), R01AG056534 (PI: A.L.J.), K24AG046373 (PI: A.L.J.), and Alzheimer’s Association IIRG-08-88733 (PI: A.L.J.). Data collection and analysis was supported by UL1TR000445, UL1TR002243 (Vanderbilt Clinical Translational Science Award), and S10OD023680 (Vanderbilt’s High-Performance Computer Cluster for Biomedical Research).

## Declaration of Competing Interest

Timothy J. Hohman, PhD is a member of the scientific advisory board for Vivid Genomics and serves on the editorial board for Alzheimer’s & Dementia and Alzheimer’s & Dementia: Translational Research and Clinical Intervention.

## Supplementary Materials

Supplementary material for this article is available with the online version here: https://doi.org/10.1162/imag_a_00293.

## Supplementary Material

Supplementary Material

Supplementary Material
