# Supplementary material for: Free-water: A promising structural biomarker for cognitive decline in aging and mild cognitive impairment
Source: Imaging Neurosci (Camb). 2024 Sep 18;2:imag-2-00293. doi: 10.1162/imag_a_00293 (PMC11540062; doi:10.1162/imag_a_00293)
Supplement: Supplementary Material [file imag_a_00293-supp.pdf]

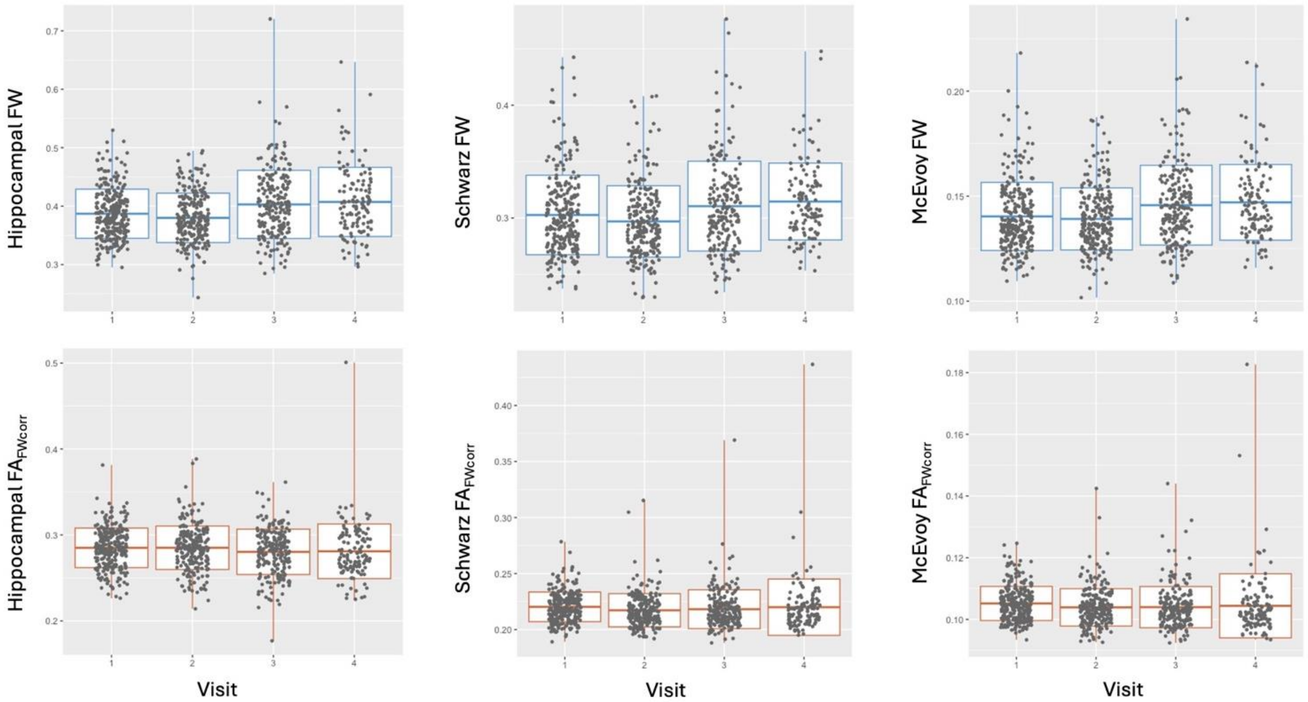

Figure S1. Boxplots showing the mean and standard deviation of FW measures at each visit.

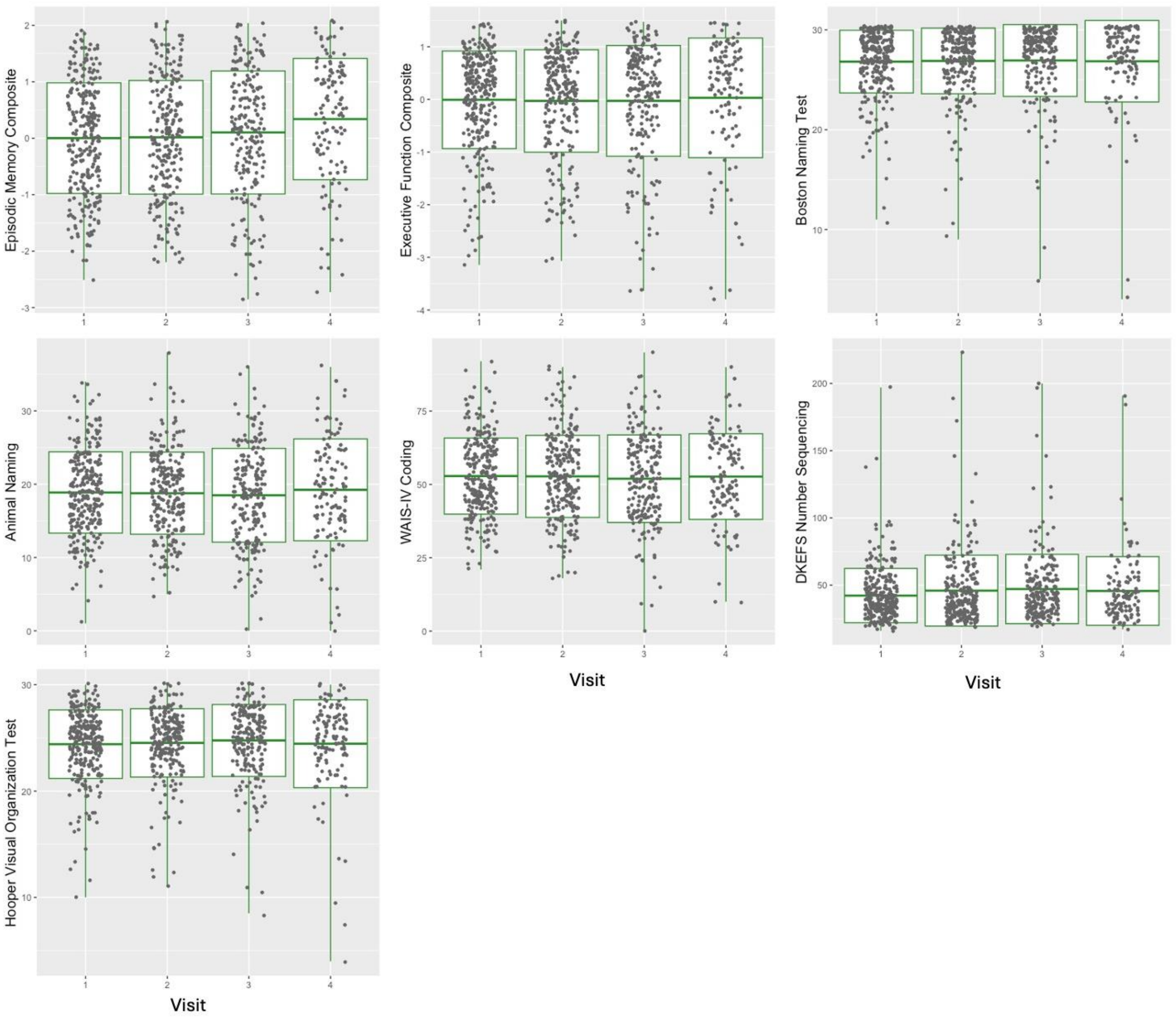

Figure S2. Boxplots showing the mean and standard deviation of cognitive test scores at each visit.

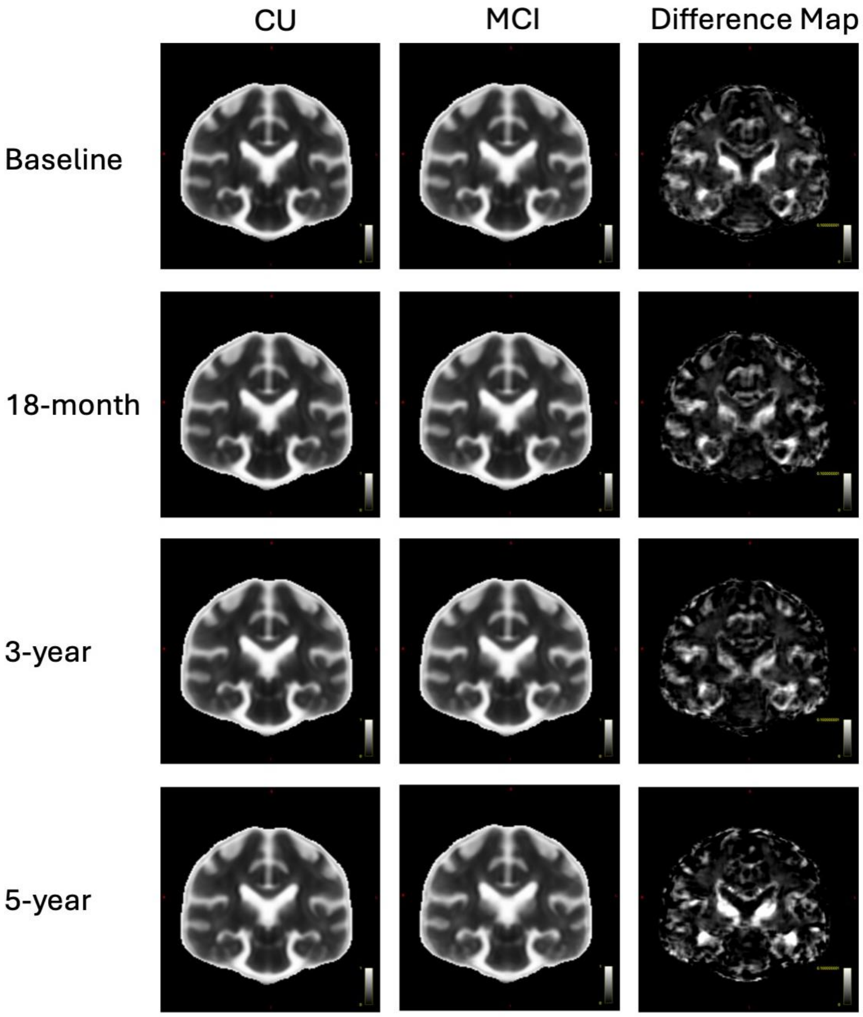

Figure S3. Average maps of FW values across all CU (column 1) and MCI (column 2) individuals and the difference between the two maps (column 3) at each study timepoint.

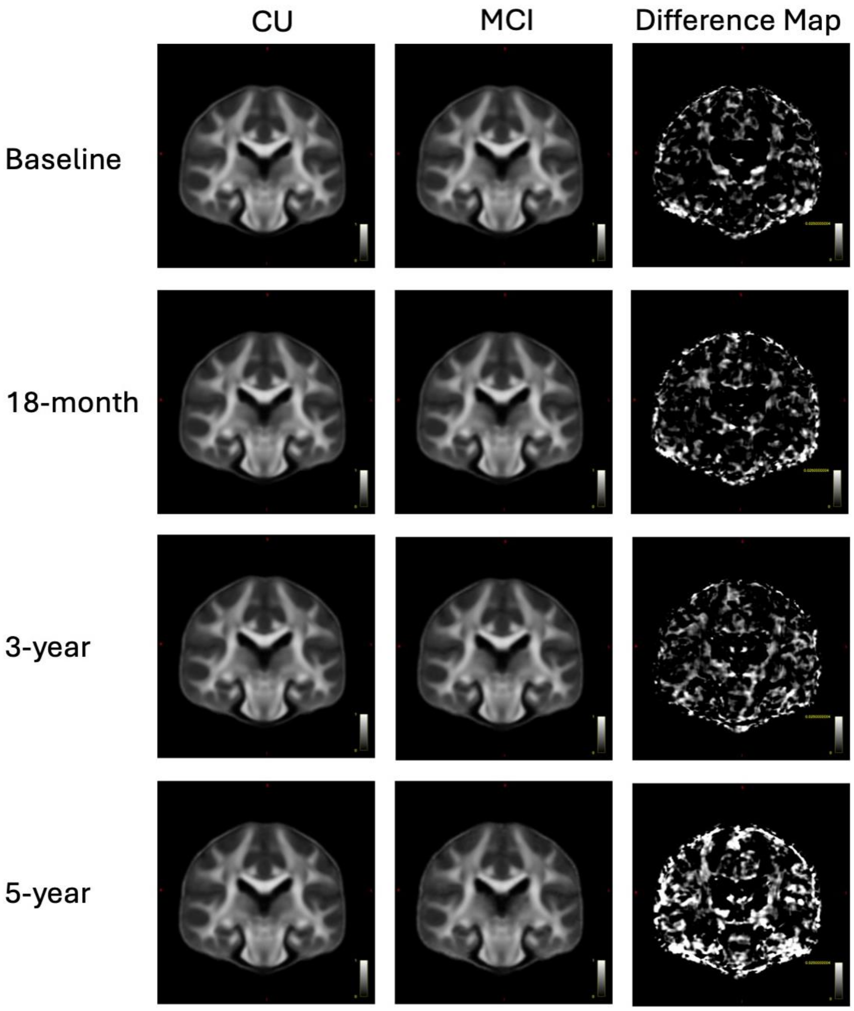

Figure S4. Average maps of FW-corrected FA values across all CU (column 1) and MCI (column 2) individuals and the difference between the two maps (column 3) at each study timepoint.

Supplemental Table 1

PREDICTOR	OUTCOME	B	SE	PVAL	TVAL	p.fdr
McEvoy AD Signature	Memory Composite	-0.64	0.22	0.004	-2.930	0.087
Schwarz AD Signature	Boston Naming Test	-44.36	14.73	0.003	-3.012	0.087
Schwarz FA(FWcorr)	Boston Naming Test	47.27	16.27	0.004	2.905	0.087
McEvoy FA(FWcorr)	Boston Naming Test	47.27	16.86	0.005	2.803	0.089
McEvoy FW	Boston Naming Test	20.74	8.07	0.011	2.569	0.141
McEvoy AD Signature	Boston Naming Test	-1.99	0.89	0.025	-2.248	0.279
Hippocampal FW	Boston Naming Test	17.18	8.28	0.039	2.077	0.284
McEvoy FW	WAIS-IV Coding	-66.42	31.97	0.039	-2.077	0.284
Schwarz FA(conventional)	Executive Function Composite	-9.21	4.35	0.035	-2.119	0.284
Schwarz AD Signature	Memory Composite	-7.22	3.70	0.052	-1.950	0.289
McEvoy FA(FWcorr)	WAIS-IV Coding	-126.86	67.15	0.060	-1.889	0.289
Schwarz FW	Boston Naming Test	16.22	8.44	0.056	1.922	0.289
McEvoy FA(conventional)	Executive Function Composite	-10.22	5.13	0.047	-1.994	0.289
Hippocampal FW(conventional)	Boston Naming Test	-22.95	12.22	0.061	-1.878	0.289
McEvoy AD Signature	Executive Function Composite	-0.42	0.23	0.067	-1.835	0.297
Schwarz FW	WAIS-IV Coding	-55.00	33.19	0.099	-1.657	0.393
Schwarz FA(conventional)	DKEFS Number Sequencing	184.46	112.18	0.101	1.644	0.393
McEvoy FA(FWcorr)	Memory Composite	6.54	4.25	0.125	1.539	0.412
Hippocampal FW(conventional)	Animal Naming Test	-31.91	20.60	0.122	-1.549	0.412
Hippocampal FW(conventional)	Executive Function Composite	-4.92	3.14	0.118	-1.566	0.412
Schwarz FA(FWcorr)	Memory Composite	6.09	4.10	0.138	1.487	0.414
Hippocampal FA(FWcorr)	Boston Naming Test	-20.25	13.41	0.132	-1.510	0.414
Schwarz AD Signature	Executive Function Composite	-5.32	3.91	0.174	-1.362	0.500
McEvoy FA(conventional)	DKEFS Number Sequencing	172.78	132.43	0.193	1.305	0.531
Hippocampal FA(FWcorr)	Animal Naming Test	-27.51	22.64	0.225	-1.215	0.595
Hippocampal FW(conventional)	DKEFS Number Sequencing	92.19	81.14	0.257	1.136	0.652
Hippocampal FW	WAIS-IV Coding	-32.61	32.64	0.319	-0.999	0.757
Schwarz FA(conventional)	Animal Naming Test	-28.32	28.50	0.321	-0.994	0.757
Schwarz AD Signature	WAIS-IV Coding	53.59	59.63	0.370	0.899	0.836
Schwarz FA(FWcorr)	WAIS-IV Coding	-57.12	64.99	0.380	-0.879	0.836
Hippocampal FA(FWcorr)	Memory Composite	2.65	3.34	0.429	0.793	0.857
Hippocampal FA(FWcorr)	DKEFS Number Sequencing	70.63	89.07	0.428	0.793	0.857
McEvoy FA(conventional)	Animal Naming Test	-28.08	33.61	0.404	-0.835	0.857
Hippocampal FA(FWcorr)	WAIS-IV Coding	37.91	52.58	0.471	0.721	0.889
Hippocampal FW	Memory Composite	1.49	2.03	0.465	0.732	0.889
Schwarz AD Signature	Animal Naming Test	-17.86	25.69	0.487	-0.695	0.894
Schwarz FW	Animal Naming Test	-9.41	14.16	0.507	-0.665	0.904
McEvoy AD Signature	Animal Naming Test	-0.66	1.53	0.666	-0.432	0.913
McEvoy AD Signature	WAIS-IV Coding	1.03	3.56	0.774	0.288	0.913
Schwarz AD Signature	DKEFS Number Sequencing	-25.76	101.49	0.800	-0.254	0.913
McEvoy FA(FWcorr)	Executive Function Composite	-2.28	4.45	0.609	-0.512	0.913
McEvoy FA(FWcorr)	DKEFS Number Sequencing	50.69	114.74	0.659	0.442	0.913
Schwarz FA(FWcorr)	Executive Function Composite	-1.85	4.29	0.666	-0.432	0.913
Hippocampal FA(FWcorr)	Executive Function Composite	-1.47	3.46	0.672	-0.424	0.913
Hippocampal FW	Animal Naming Test	5.16	14.02	0.713	0.368	0.913
Hippocampal FW	Executive Function Composite	-0.54	2.14	0.802	-0.251	0.913
Hippocampal FW	DKEFS Number Sequencing	16.24	55.44	0.770	0.293	0.913
McEvoy FW	Animal Naming Test	-7.29	13.66	0.594	-0.534	0.913
McEvoy FW	Executive Function Composite	-1.04	2.10	0.621	-0.494	0.913
McEvoy FW	Memory Composite	0.90	2.01	0.654	0.448	0.913
McEvoy FW	DKEFS Number Sequencing	14.88	54.53	0.785	0.273	0.913
Schwarz FW	Executive Function Composite	-0.55	2.17	0.801	-0.253	0.913
Schwarz FW	DKEFS Number Sequencing	-21.19	56.41	0.707	-0.376	0.913
McEvoy FA(conventional)	Boston Naming Test	-5.04	19.95	0.801	-0.253	0.913
McEvoy FA(conventional)	WAIS-IV Coding	-41.87	78.08	0.592	-0.536	0.913
Schwarz FA(conventional)	Memory Composite	1.29	4.20	0.760	0.306	0.913
Hippocampal FW(conventional)	WAIS-IV Coding	-21.09	48.07	0.661	-0.439	0.913
Hippocampal FW(conventional)	Memory Composite	-1.87	3.05	0.540	-0.614	0.913
Schwarz FW	Memory Composite	0.40	2.10	0.850	0.189	0.935
McEvoy FA(conventional)	Memory Composite	-0.97	4.95	0.845	-0.195	0.935
McEvoy FA(FWcorr)	Animal Naming Test	3.02	28.97	0.917	0.104	0.947
Schwarz FA(FWcorr)	Animal Naming Test	-2.87	27.87	0.918	-0.103	0.947
Schwarz FA(conventional)	Boston Naming Test	1.85	16.94	0.913	0.109	0.947
Schwarz FA(conventional)	WAIS-IV Coding	8.77	66.24	0.895	0.132	0.947
McEvoy AD Signature	DKEFS Number Sequencing	0.25	6.06	0.968	0.041	0.983
Schwarz FA(FWcorr)	DKEFS Number Sequencing	2.01	110.46	0.986	0.018	0.986
McEvoy AD Signature	Hooper Visual Organization	-1.93	0.93	0.039	-2.074	0.255
Schwarz AD Signature	Hooper Visual Organization	-26.48	15.70	0.093	-1.687	0.255
McEvoy FA(FWcorr)	Hooper Visual Organization	30.98	17.54	0.078	1.766	0.255
Schwarz FA(FWcorr)	Hooper Visual Organization	30.15	16.82	0.074	1.793	0.255
Hippocampal FA(FWcorr)	Hooper Visual Organization	-6.36	13.97	0.649	-0.456	0.715
Hippocampal FW	Hooper Visual Organization	6.32	8.60	0.463	0.735	0.715
McEvoy FW	Hooper Visual Organization	5.73	8.47	0.500	0.676	0.715
Schwarz FW	Hooper Visual Organization	2.42	8.78	0.783	0.276	0.783
McEvoy FA(conventional)	Hooper Visual Organization	13.91	20.60	0.500	0.675	0.715
Schwarz FA(conventional)	Hooper Visual Organization	9.70	17.45	0.579	0.556	0.715
Hippocampal FA(conventional)	Hooper Visual Organization	-5.80	12.75	0.650	-0.455	0.715

Supplemental Table 2

PREDICTOR	B	SE	PVAL	TVAL	p.fdr
Schwarz AD Signature	2.27	0.06	0	40.81	0
Schwarz FA(FWcorr)	0.21	0.01	0	41.33	0
McEvoy FA(FWcorr)	0.10	0.00	0	49.84	0
Hippocampal FA(FWcorr)	0.29	0.01	8.27E-196	29.85	1.86E-195
McEvoy FW	0.15	0.01	3.16E-156	26.63	5.70E-156
Schwarz FW	0.31	0.01	7.56E-148	25.90	1.13E-147
Hippocampal FW	0.41	0.02	5.47E-140	25.19	7.03E-140
Hippocampal Volume	5447.35	337.37	1.20E-58	16.15	1.35E-58
McEvoy AD Signature	-0.84	1.15	0.46	-0.74	0.46

Supplemental Table 3

PREDICTOR	OUTCOME	B	SE	PVAL	TVAL	p.fdr
Hippocampal FW	Memory Composite	-2.98	0.42	1.24E-12	-7.10	8.91E-11
McEvoy AD Signature	Memory Composite	0.25	0.04	3.32E-10	6.28	1.20E-08
Schwarz AD Signature	Memory Composite	4.22	0.69	7.24E-10	6.16	1.74E-08
McEvoy AD Signature	Boston Naming Test	0.94	0.16	4.96E-09	5.85	8.93E-08
Schwarz AD Signature	Boston Naming Test	15.61	2.74	1.20E-08	5.70	1.73E-07
McEvoy FW	Memory Composite	-2.33	0.42	2.17E-08	-5.60	2.61E-07
Hippocampal Volume	Memory Composite	1.47	0.30	9.65E-07	4.90	9.92E-06
Hippocampal FW	Animal Naming Test	-12.97	2.79	3.47E-06	-4.64	3.02E-05
Hippocampal FW	Executive Function Composite	-2.08	0.45	3.77E-06	-4.62	3.02E-05
McEvoy AD Signature	Animal Naming Test	1.20	0.26	4.90E-06	4.57	3.53E-05
McEvoy AD Signature	Executive Function Composite	0.19	0.04	6.88E-06	4.50	3.94E-05
McEvoy FW	Animal Naming Test	-12.39	2.76	7.11E-06	-4.49	3.94E-05
Schwarz FW	Memory Composite	-1.80	0.40	7.11E-06	-4.49	3.94E-05
Schwarz FW	Animal Naming Test	-10.42	2.63	7.32E-05	-3.97	3.76E-04
Schwarz AD Signature	Executive Function Composite	2.79	0.74	1.55E-04	3.78	7.01E-04
Schwarz FW	Executive Function Composite	-1.57	0.42	1.56E-04	-3.78	7.01E-04
Schwarz AD Signature	Animal Naming Test	16.59	4.53	2.52E-04	3.66	1.01E-03
Hippocampal FA(conventional)	Animal Naming Test	11.67	3.19	2.53E-04	3.66	1.01E-03
Hippocampal Volume	Animal Naming Test	7.03	1.94	3.00E-04	3.62	1.14E-03
McEvoy FW	Executive Function Composite	-1.55	0.44	4.06E-04	-3.54	1.46E-03
Hippocampal FA(conventional)	Executive Function Composite	1.85	0.52	4.29E-04	3.52	1.47E-03
Hippocampal FW	Boston Naming Test	-5.89	1.71	5.77E-04	-3.44	1.89E-03
Hippocampal FA(conventional)	Memory Composite	1.74	0.51	6.04E-04	3.43	1.89E-03
Hippocampal Volume	Boston Naming Test	4.04	1.19	6.59E-04	3.41	1.98E-03
Schwarz FA(FWcorr)	Boston Naming Test	-9.25	2.78	8.61E-04	-3.33	2.48E-03
McEvoy FA(FWcorr)	Boston Naming Test	-10.60	3.22	1.01E-03	-3.29	2.79E-03
McEvoy FW	Boston Naming Test	-5.18	1.63	1.51E-03	-3.17	4.04E-03
McEvoy FA(FWcorr)	Memory Composite	-2.59	0.84	2.13E-03	-3.07	5.47E-03
Schwarz FW	DKEFS Number Sequencing	27.93	9.49	3.25E-03	2.94	8.07E-03
Schwarz FA(FWcorr)	Memory Composite	-2.05	0.73	4.68E-03	-2.83	1.12E-02
Schwarz FW	Boston Naming Test	-4.35	1.56	5.45E-03	-2.78	1.27E-02
Schwarz FA(FWcorr)	Executive Function Composite	-1.95	0.75	9.13E-03	-2.61	2.05E-02
Hippocampal Volume	Executive Function Composite	0.79	0.32	1.26E-02	2.49	2.76E-02
Hippocampal FW	DKEFS Number Sequencing	25.63	10.36	1.34E-02	2.47	2.83E-02
Hippocampal Volume	WAIS-IV Coding	11.32	4.73	1.67E-02	2.39	3.44E-02
McEvoy FW	DKEFS Number Sequencing	23.74	10.17	1.96E-02	2.33	3.91E-02
McEvoy FA(FWcorr)	Executive Function Composite	-2.01	0.87	2.06E-02	-2.31	3.94E-02
Hippocampal FA(conventional)	WAIS-IV Coding	18.11	7.87	2.13E-02	2.30	3.94E-02
Hippocampal FA(conventional)	DKEFS Number Sequencing	-26.69	11.56	2.10E-02	-2.31	3.94E-02
Hippocampal FA(FWcorr)	Animal Naming Test	7.34	3.37	2.93E-02	2.18	5.27E-02
Schwarz FA(FWcorr)	Animal Naming Test	-10.08	4.66	3.07E-02	-2.16	5.39E-02
Hippocampal FA(FWcorr)	Executive Function Composite	1.08	0.54	4.40E-02	2.01	7.55E-02
Schwarz FA(FWcorr)	DKEFS Number Sequencing	31.61	16.21	5.12E-02	1.95	8.58E-02
Hippocampal FA(FWcorr)	DKEFS Number Sequencing	-22.09	11.90	6.33E-02	-1.86	1.04E-01
McEvoy FA(FWcorr)	Animal Naming Test	-9.81	5.45	7.21E-02	-1.80	1.15E-01
Hippocampal FA(FWcorr)	WAIS-IV Coding	14.08	8.16	8.45E-02	1.73	1.32E-01
Hippocampal FA(conventional)	Boston Naming Test	3.38	2.02	9.44E-02	1.67	1.45E-01
Schwarz AD Signature	DKEFS Number Sequencing	-26.65	16.72	1.11E-01	-1.59	1.67E-01
McEvoy AD Signature	DKEFS Number Sequencing	-1.54	0.98	1.15E-01	-1.58	1.69E-01
Hippocampal FW	WAIS-IV Coding	-10.51	6.93	1.29E-01	-1.52	1.86E-01
McEvoy AD Signature	WAIS-IV Coding	0.94	0.66	1.54E-01	1.43	2.17E-01
McEvoy FA(conventional)	Memory Composite	1.02	0.78	1.91E-01	1.31	2.64E-01
Schwarz FW	WAIS-IV Coding	-8.19	6.35	1.97E-01	-1.29	2.67E-01
McEvoy FW	WAIS-IV Coding	-8.55	6.69	2.02E-01	-1.28	2.69E-01
McEvoy FA(conventional)	Animal Naming Test	5.92	4.95	2.32E-01	1.20	3.04E-01
McEvoy FA(FWcorr)	DKEFS Number Sequencing	21.89	19.05	2.50E-01	1.15	3.22E-01
Schwarz AD Signature	WAIS-IV Coding	11.80	11.13	2.89E-01	1.06	3.65E-01
Schwarz FA(FWcorr)	WAIS-IV Coding	-11.04	11.42	3.33E-01	-0.97	4.14E-01
Hippocampal FA(FWcorr)	Memory Composite	0.42	0.53	4.23E-01	0.80	5.17E-01
McEvoy FA(conventional)	DKEFS Number Sequencing	-13.38	17.47	4.44E-01	-0.77	5.32E-01
Hippocampal Volume	DKEFS Number Sequencing	-5.22	7.16	4.66E-01	-0.73	5.50E-01
McEvoy FA(conventional)	Executive Function Composite	0.58	0.81	4.77E-01	0.71	5.54E-01
Schwarz FA(conventional)	DKEFS Number Sequencing	-9.79	15.14	5.18E-01	-0.65	5.92E-01
McEvoy FA(FWcorr)	WAIS-IV Coding	-8.35	13.27	5.29E-01	-0.63	5.95E-01
Schwarz FA(conventional)	Memory Composite	0.41	0.68	5.47E-01	0.60	6.06E-01
Schwarz FA(conventional)	Animal Naming Test	1.98	4.31	6.45E-01	0.46	7.04E-01
Schwarz FA(conventional)	Boston Naming Test	-1.17	2.65	6.59E-01	-0.44	7.08E-01
Schwarz FA(conventional)	Executive Function Composite	0.27	0.71	7.04E-01	0.38	7.45E-01
McEvoy FA(conventional)	WAIS-IV Coding	3.49	12.11	7.73E-01	0.29	8.07E-01
Hippocampal FA(FWcorr)	Boston Naming Test	-0.52	2.09	8.01E-01	-0.25	8.24E-01
McEvoy FA(conventional)	Boston Naming Test	-0.30	3.08	9.21E-01	-0.10	9.34E-01
Schwarz FA(conventional)	WAIS-IV Coding	0.57	10.50	9.56E-01	0.05	9.56E-01
McEvoy AD Signature	Hooper Visual Organization	0.68	0.17	7.61E-05	3.96	3.04E-04
Schwarz AD Signature	Hooper Visual Organization	10.90	2.95	2.24E-04	3.69	4.87E-04
McEvoy FA(FWcorr)	Hooper Visual Organization	-12.54	3.42	2.43E-04	-3.67	4.87E-04
Schwarz FA(FWcorr)	Hooper Visual Organization	-12.37	2.91	2.16E-05	-4.25	1.30E-04
Hippocampal FA(FWcorr)	Hooper Visual Organization	2.56	2.18	2.40E-01	1.17	2.89E-01
Hippocampal FW	Hooper Visual Organization	-8.18	1.80	5.73E-06	-4.54	6.88E-05
Hippocampal Volume	Hooper Visual Organization	4.77	1.26	1.53E-04	3.79	4.58E-04
McEvoy FW	Hooper Visual Organization	-6.25	1.76	3.75E-04	-3.56	5.63E-04
Schwarz FW	Hooper Visual Organization	-5.93	1.66	3.59E-04	-3.57	5.63E-04
McEvoy FA(conventional)	Hooper Visual Organization	-1.80	3.21	5.74E-01	-0.56	5.74E-01
Schwarz FA(conventional)	Hooper Visual Organization	-2.67	2.76	3.33E-01	-0.97	3.63E-01
Hippocampal FA(conventional)	Hooper Visual Organization	5.13	2.10	1.47E-02	2.44	1.95E-02

Supplemental Table 4

PREDICTOR	OUTCOME	B	SE	PVAL	TVAL	p.fdr
Schwarz FA(FWcorr)	Boston Naming Test	42.22	1.66	2.32E-143	25.49	2.09E-142
McEvoy FA(FWcorr)	Boston Naming Test	44.87	1.87	9.51E-128	24.04	4.28E-127
McEvoy FW	Boston Naming Test	39.15	1.68	2.87E-120	23.32	8.60E-120
McEvoy AD Signature	Boston Naming Test	26.90	1.17	6.52E-117	22.99	1.47E-116
Schwarz FW	Boston Naming Test	37.65	1.69	3.88E-110	22.30	6.98E-110
Hippocampal FW	Boston Naming Test	34.02	1.60	6.94E-100	21.21	1.04E-99
McEvoy FW	Hooper Visual Organization	34.15	1.79	2.34E-81	19.10	2.11E-80
Schwarz FA(FWcorr)	Hooper Visual Organization	34.07	1.83	2.49E-77	18.61	1.12E-76
Hippocampal FW	Hooper Visual Organization	31.58	1.71	2.85E-76	18.48	8.54E-76
McEvoy AD Signature	Hooper Visual Organization	23.08	1.25	8.54E-76	18.42	1.92E-75
McEvoy FA(FWcorr)	Hooper Visual Organization	37.15	2.04	6.70E-74	18.19	1.21E-73
Schwarz FW	Hooper Visual Organization	32.61	1.81	9.32E-73	18.04	1.40E-72
Hippocampal FA(FWcorr)	Boston Naming Test	28.70	1.94	2.52E-49	14.76	3.24E-49
Hippocampal FA(FWcorr)	Hooper Visual Organization	24.01	2.09	1.89E-30	11.47	2.44E-30
McEvoy FW	Animal Naming Test	31.13	2.93	2.11E-26	10.63	1.90E-25
Schwarz FW	Animal Naming Test	29.92	2.99	1.34E-23	10.01	6.01E-23
Hippocampal Volume	Boston Naming Test	16.98	1.73	8.54E-23	9.83	9.60E-23
Hippocampal Volume	Memory Composite	-4.14	0.42	6.59E-23	-9.85	6.59E-22
Hippocampal FW	Animal Naming Test	26.78	2.86	6.68E-21	9.38	2.00E-20
Hippocampal Volume	Executive Function Composite	-4.04	0.45	1.50E-19	-9.04	1.50E-18
McEvoy FW	WAIS-IV Coding	55.76	6.62	3.50E-17	8.43	2.35E-16
Hippocampal FW	WAIS-IV Coding	52.43	6.26	5.22E-17	8.38	2.35E-16
Schwarz FW	WAIS-IV Coding	52.95	6.64	1.60E-15	7.97	4.81E-15
Schwarz AD Signature	Memory Composite	-5.30	0.72	1.40E-13	-7.40	6.99E-13
Hippocampal Volume	Hooper Visual Organization	11.98	1.79	2.03E-11	6.70	2.28E-11
McEvoy FA(FWcorr)	Animal Naming Test	24.66	3.67	1.73E-11	6.73	3.90E-11
Hippocampal Volume	DKEFS Number Sequencing	73.40	10.66	5.72E-12	6.89	5.15E-11
Schwarz FA(FWcorr)	Animal Naming Test	21.56	3.25	3.25E-11	6.63	5.85E-11
McEvoy AD Signature	Animal Naming Test	12.38	1.91	9.91E-11	6.47	1.49E-10
McEvoy AD Signature	DKEFS Number Sequencing	45.25	6.87	4.42E-11	6.59	1.99E-10
McEvoy AD Signature	WAIS-IV Coding	30.15	4.90	7.69E-10	6.15	1.73E-09
Schwarz AD Signature	Executive Function Composite	-4.77	0.77	5.02E-10	-6.22	2.51E-09
McEvoy FA(FWcorr)	WAIS-IV Coding	43.88	8.74	5.22E-07	5.02	9.39E-07
McEvoy FW	Executive Function Composite	2.22	0.44	4.11E-07	5.06	1.37E-06
Schwarz FA(FWcorr)	WAIS-IV Coding	37.81	8.35	5.92E-06	4.53	8.87E-06
Hippocampal FA(FWcorr)	DKEFS Number Sequencing	61.20	14.25	1.75E-05	4.29	5.24E-05
Schwarz FW	Executive Function Composite	1.84	0.44	3.40E-05	4.15	6.79E-05
McEvoy AD Signature	Memory Composite	-1.16	0.29	5.03E-05	-4.05	1.68E-04
Schwarz AD Signature	DKEFS Number Sequencing	81.48	20.70	8.27E-05	3.94	1.86E-04
Hippocampal FA(FWcorr)	WAIS-IV Coding	27.13	7.22	1.72E-04	3.76	2.22E-04
Hippocampal FW	Executive Function Composite	1.50	0.42	3.06E-04	3.61	4.73E-04
McEvoy AD Signature	Executive Function Composite	-1.08	0.30	3.31E-04	-3.59	4.73E-04
Schwarz AD Signature	Boston Naming Test	9.62	2.76	4.91E-04	3.49	4.91E-04
Hippocampal FA(FWcorr)	Executive Function Composite	-1.60	0.53	2.37E-03	-3.04	2.97E-03
McEvoy FW	Memory Composite	1.31	0.42	1.94E-03	3.10	3.88E-03
Hippocampal FA(FWcorr)	Memory Composite	-1.24	0.50	1.25E-02	-2.50	2.08E-02
Schwarz FW	Memory Composite	0.97	0.43	2.55E-02	2.23	3.64E-02
Hippocampal FA(FWcorr)	Animal Naming Test	7.09	3.49	4.19E-02	2.03	5.38E-02
McEvoy FA(FWcorr)	Executive Function Composite	1.04	0.53	5.02E-02	1.96	5.58E-02
Hippocampal FW	Memory Composite	0.76	0.41	6.26E-02	1.86	7.83E-02
McEvoy FA(FWcorr)	Memory Composite	0.87	0.50	8.09E-02	1.75	8.99E-02
Schwarz AD Signature	Animal Naming Test	-7.57	5.18	1.44E-01	-1.46	1.62E-01
Schwarz AD Signature	Hooper Visual Organization	4.02	3.00	1.81E-01	1.34	1.81E-01
Schwarz FA(FWcorr)	Executive Function Composite	0.48	0.47	3.09E-01	1.02	3.09E-01
Schwarz FA(FWcorr)	Memory Composite	0.39	0.45	3.88E-01	0.86	3.88E-01
Hippocampal Volume	Animal Naming Test	-2.13	2.85	4.55E-01	-0.75	4.55E-01
Hippocampal FW	DKEFS Number Sequencing	-10.95	11.32	3.33E-01	-0.97	6.00E-01
McEvoy FW	DKEFS Number Sequencing	-9.58	11.91	4.21E-01	-0.80	6.32E-01
Hippocampal Volume	WAIS-IV Coding	-1.60	6.71	8.12E-01	-0.24	8.27E-01
Schwarz AD Signature	WAIS-IV Coding	2.34	10.69	8.27E-01	0.22	8.27E-01
Schwarz FW	DKEFS Number Sequencing	-4.49	11.88	7.05E-01	-0.38	9.07E-01
Schwarz FA(FWcorr)	DKEFS Number Sequencing	-1.61	18.25	9.30E-01	-0.09	9.95E-01
McEvoy FA(FWcorr)	DKEFS Number Sequencing	0.13	19.78	9.95E-01	0.01	9.95E-01
